# Gypsum endolithic phototrophs under moderate climate (Southern Sicily): their diversity and pigment composition

**DOI:** 10.3389/fmicb.2023.1175066

**Published:** 2023-07-06

**Authors:** Kateřina Němečková, Jan Mareš, Lenka Procházková, Adam Culka, Filip Košek, Jacek Wierzchos, Linda Nedbalová, Jan Dudák, Veronika Tymlová, Jan Žemlička, Andreja Kust, Jan Zima, Eva Nováková, Jan Jehlička

**Affiliations:** ^1^Institute of Geochemistry, Mineralogy and Mineral Resources, Faculty of Science, Charles University, Prague, Czechia; ^2^Institute of Hydrobiology, Biology Centre of the Czech Academy of Sciences, České Budějovice, Czechia; ^3^Center Algatech, Institute of Microbiology, The Czech Academy of Sciences, Třeboň, Czechia; ^4^Department of Ecology, Faculty of Science, Charles University, Prague, Czechia; ^5^Department of Biochemistry and Microbial Ecology, Museo Nacional de Ciencias Naturales - Consejo Superior de Investigaciones Científicas, Madrid, Spain; ^6^Institute of Experimental and Applied Physics, Czech Technical University in Prague, Prague, Czechia; ^7^Department of Earth and Planetary Science, University of Berkeley, Berkeley, CA, United States; ^8^Innovative Genomics Institute, University of California, Berkeley, Berkeley, CA, United States; ^9^Department of Parasitology, Faculty of Science, University of South Bohemia, České Budějovice, Czechia

**Keywords:** endoliths, gypsum, phototrophs, cyanobacteria, green algae, metagenomics

## Abstract

In this study, we used microscopic, spectroscopic, and molecular analysis to characterize endolithic colonization in gypsum (selenites and white crystalline gypsum) from several sites in Sicily. Our results showed that the dominant microorganisms in these environments are cyanobacteria, including: *Chroococcidiopsis* sp., *Gloeocapsopsis pleurocapsoides*, *Gloeocapsa compacta*, and *Nostoc* sp., as well as orange pigmented green microalgae from the *Stephanospherinia* clade. Single cell and filament sequencing coupled with 16S rRNA amplicon metagenomic profiling provided new insights into the phylogenetic and taxonomic diversity of the endolithic cyanobacteria. These organisms form differently pigmented zones within the gypsum. Our metagenomic profiling also showed differences in the taxonomic composition of endoliths in different gypsum varieties. Raman spectroscopy revealed that carotenoids were the most common pigments present in the samples. Other pigments such as gloeocapsin and scytonemin were also detected in the near-surface areas, suggesting that they play a significant role in the biology of endoliths in this environment. These pigments can be used as biomarkers for basic taxonomic identification, especially in case of cyanobacteria. The findings of this study provide new insights into the diversity and distribution of phototrophic microorganisms and their pigments in gypsum in Southern Sicily. Furthemore, this study highlights the complex nature of endolithic ecosystems and the effects of gypsum varieties on these communities, providing additional information on the general bioreceptivity of these environments.

## 1. Introduction

Rock substrates are used as habitats by various microorganisms all over the globe (Gorbushina, 2007). These organisms are known as lithobionts and can be divided into several groups—epiliths (inhabiting the surface of rocks), endoliths (inhabiting inner parts of rocks close to the rock’s surface), hypoliths (inhabiting spaces below rocks) and hypoendoliths (inhabiting inner part of the rocks, close to the rock’s bottom). Endoliths can further be divided into chasmoendoliths (colonizing rock fissures and cracks) and cryptoendoliths (colonizing the pores within rocks). Lithobionts represent a diverse group of organisms—microalgae, lichens, fungi, cyanobacteria, heterotrophic bacteria, and archaea (Golubic et al., 1981). These rock-dwelling communities are significant in many research areas such as the ecology of extreme environments (Friedmann, 1982), astrobiology (Vítek et al., 2014a), cultural heritage (Sanmartín et al., 2018), or even radioactive waste disposal (Pitonzo et al., 1999).

Generally, endolithic environments provide protection against changing exterior conditions such as solar radiation, temperature, water availability, etc. (Golubic et al., 1981; Walker and Pace, 2007). Despite that, not all rock-substrates are suitable for lithic growth. The capacity to support microbial growth is determined by its bioreceptivity, i.e., the ability of substrates to be colonized. Bioreceptivity is influenced by both intrinsic (roughness, pore size distribution, mineral composition and transparency, among other properties) and extrinsic factors (climatic conditions, availability of nutrients, composition of lithobiontic communities, synergism etc.) (Walker and Pace, 2007). Sedimentary rocks (e.g., sandstones (Archer et al., 2017), limestones (Wong et al., 2010), halite (Wierzchos et al., 2006) and gypsum (Culka et al., 2014)) are among the most common lithic substrates. Nevertheless, pyroclastic, magmatic or metamorphic rocks are suitable lithic habitats as well (e.g., ignimbrite or granites) (Crits-Christoph et al., 2016; Vítek et al., 2017).

Cyanobacteria are frequently found in these lithic habitats (primarily dominated by *Chroococcidiopsis* spp.), and thus the rock-substrates must allow sufficient penetration of light for photosynthesis (Walker and Pace, 2007). In many cases, photosynthetic and UV-screening pigments (e.g., carotenoids and scytonemin) produced by microbial communities dominated by cyanobacteria manifest themselves as colorful spots or lines on rocks; such phenomenon being known as “Tintenstriche” (Whitton, 2012). These has been found in various regions around the Earth (e.g., in the Atacama desert, Crits-Christoph et al., 2016 and Dry Valleys of Antarctica, Archer et al., 2017).

Exact phylogenetic determinations of the cyanobacterial and algal species in endolithic communities can be problematic, as the morphological differences are often insignificant. Currently, the best option for identification is a polyphasic approach. This approach is based on molecular criteria (e.g., 16S rRNA/18S rDNA sequencing) complemented by other features (e.g., morphology, ecology, and ecophysiology) (Komárek, 2016; Nedbalová et al., 2017; Procházková et al., 2019). For microbial profiling of the environmental samples, culture-independent methods such as metagenomics are the most suitable ones. For example, Meslier et al. (2018) used 16S rDNA amplicon high-throughput sequencing to analyze endolithic communities, and showed variances of the relative abundances in different substrates (e.g., granite, and gypsum).

Our study area, the southern region of Sicily, is characterized by easily accessible gypsum outcrops, which were formed during the Messinian salinity crisis (Basilone, 2018). The Messinian salinity crisis, also known as the Messinian event, was a geological period approximately 5 million years ago when the Mediterranean Sea went through a period of severe drying and desiccation (Basilone, 2018). The crisis had a significant impact on the geology of the region, leading to the formation of extensive evaporite deposits, including gypsum, which are still found in Sicily today. The Messinian evaporitic succession is mainly found in the central and southern parts of Sicily.

Relevant to our study, gypsum occurs as crystals of different habitus, e.g., tabular, prismatic, saber-shaped crystals. Based on their morphology and physical properties, the different varieties of gypsum can be distinguished. Transparent gypsum crystals with perfect cleavage are known as selenites. Alabaster is a massive and fine-grained gypsum variety. In scanning electron microscopy (SEM) images, selenitic crystals are uniform in shape (of different sizes), while alabastrine crystals are irregular-subhedral (Al-Wakeel, 2002).

Our previous study on gypsum endoliths from the Torre Salsa seaside in Southern Sicily, already had revealed the presence of various cyanobacteria in investigated stony specimens (e.g., *Gloeocapsa compacta* and *Nostoc* sp.) (Jehlička et al., 2019). The analysis of different layers of colonized gypsum using Raman microspectrometry revealed presence of the different UV-screening pigments such as scytonemin and gloeocapsin in those colonizations closer to the surface; while colonizations below these layers contained only carotenoids. Other photosynthesizing pigments (e.g., chlorophylls or phycobiliproteins) were also detected (Němečková et al., 2020, 2022).

Here, we used molecular methods together with Raman microspectrometry and fluorescence microscopy in order to investigate the taxonomic composition of the endolithic communities (with a major focus on phototrophs) within the context of gypsum habitats at four sites in semi-arid Sicily: Siculiana Marina, Santa Elisabetta, Monte Perrera and Monte Gibliscemi. We also studied changes in the diversity within gypsum with different physical features. To our knowledge, this is the first study dealing with the microbial profiling of endoliths in varieties of the same substrate.

## 2. Methods

### 2.1. Sampling sites

**Figure 1:**
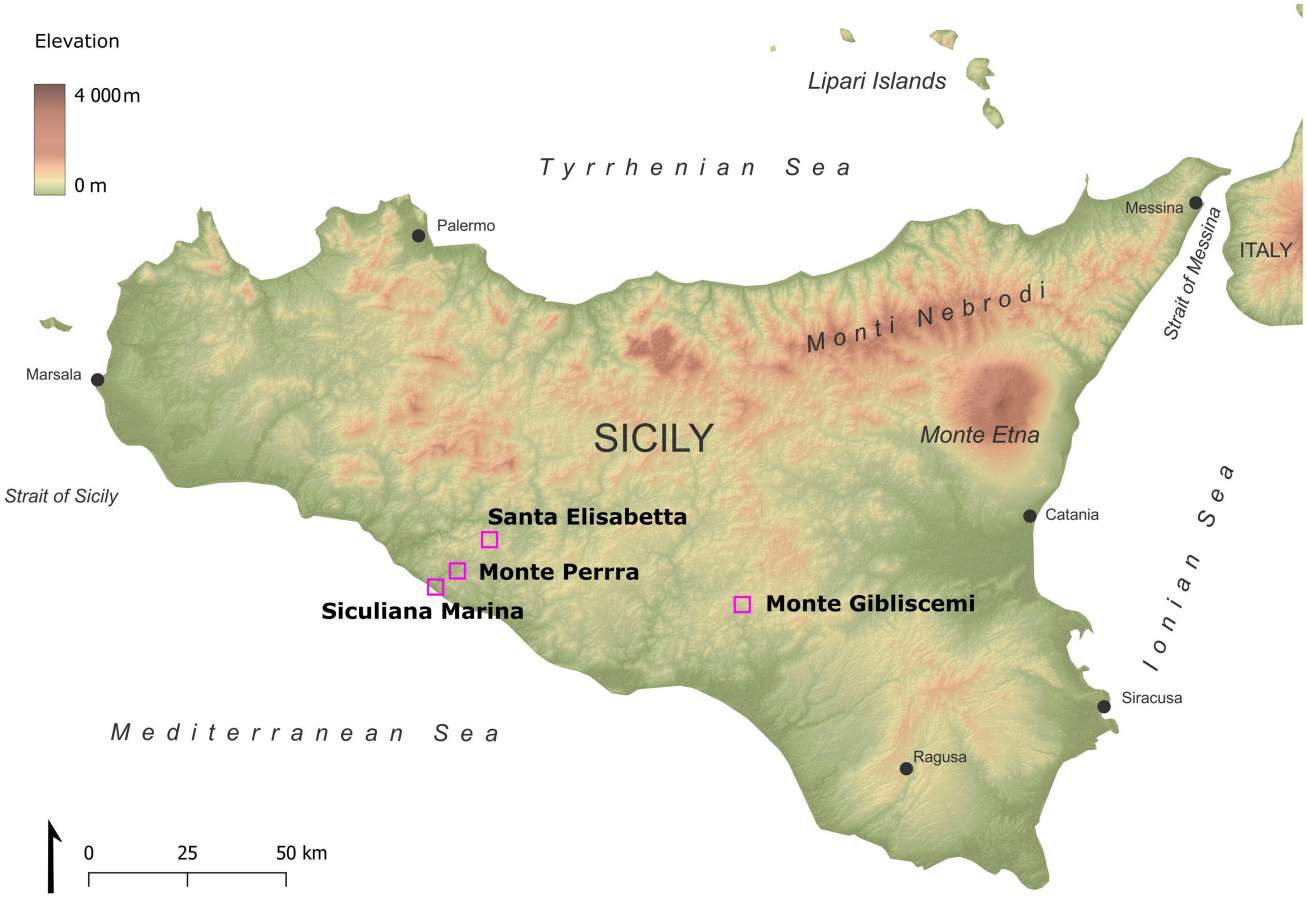
Map of studied sites in Sicily.

The sites investigated were located in the Caltanissetta Basin in Sicily (Figure 1 and Table 1). Geologically, this area is formed by a sedimentary series, which include evaporites. The Caltanissetta Basin is composed of three stratigraphic units: salt deposits, a lower gypsum unit, and an upper gypsum unit (García-Veigas et al., 2018). The lower gypsum unit is further divided into primary and resedimented gypsum. The primary gypsum includes selenites, organic-rich shales, and dolomite rocks, while the resedimented component consists of halite, brecciated limestone, gypsum cumulate deposits, allochthonous selenites, and clastic gypsum. The upper gypsum unit is made up of gypsum interbedded with clastics (Caruso et al., 2015).

**Table 1:** Sampling sites and their basic characteristics.

Site characterization					Micro-CT scans characterization		
Site name		GPS	Characterization	Lithology	Dimensions (mm)	Weathered state	Void space (~porosity)
Siculiana Marina (SM)	1S	37.3388700 N, 13.3967000 E	Sites 1S and 1A are located near road to Siculiana Marina. Samples were collected from gypsum at western side of the outcrop.	Flat “honey-color” selenitic crystals (~5–10 cm in length) in aggregates	20 × 10 × 5	Almost uncorroded with secondary crust	0.8% (uncorroded part) 17.5% (crust)
	1A			White crystalline crystals (~ 10 cm in length) in aggregates	20 × 15 × 8	Almost uncorroded with secondary crust	1.2% (uncorroded part) 15.1% (crust)
Santa Elisabetta (SE)	2	37.4472489 N, 13.5474772 E	Gypsum outcrops at western side of Santa Elisabetta Mountain	Flat “honey-color” selenitic crystals (~15–20 cm in length) in aggregates	25 × 13 × 4	Uncorroded	1.8%
Monte Perrera (MP)	3	37.3807706 N, 13.4527739 E	Gypsum outcrops at south-east side of Monte Perrera	White hard course-grain aggregate gypsum crust with fine-grained matrix underneath, with presence of cracks	10 × 10 × 5	Partially corroded with secondary crust	14.8% (corroded part) 23.4% (crust)
					15 × 10 × 10	Deeply corroded with secondary crust	16.4%
Monte Gibliscemi (MG)	4	37.2094297 N, 14.2623708 E	Blocks of gypsum located at western side of Monte Gibliscemi	Coarse-grain aggregate	23 × 10 × 10	Uncorroded	8.1%

**Figure 2:**
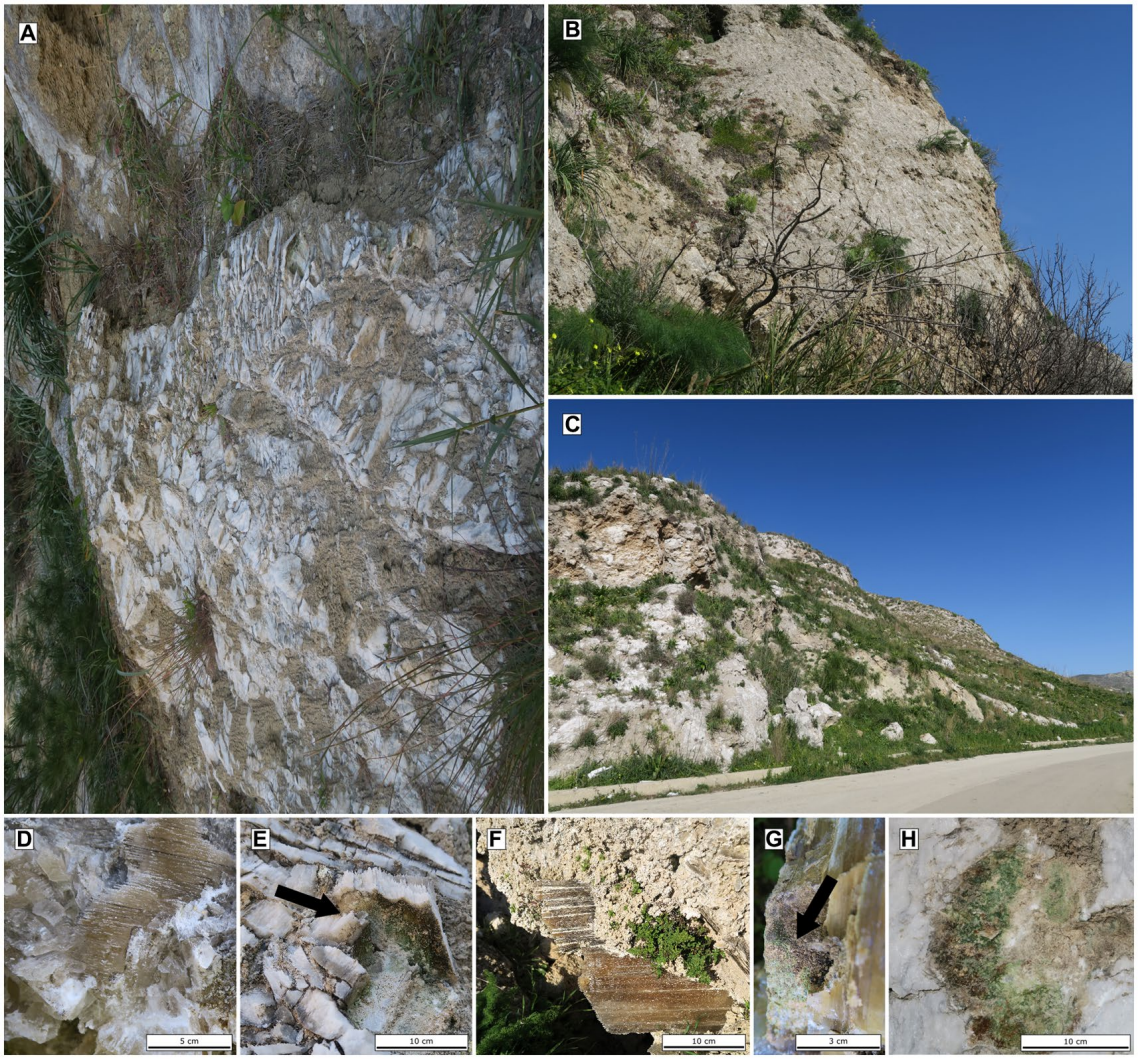
Images of gypsum outcrops at Siciliana Marina **(A)**, Santa Elisabetta **(B)** and Monte Perrera **(C)**, Detailed images of different gypsum habitats: selenites **(D)** and white crystalline-gypsum **(E)** at Siculiana Marina; selenites at Santa Elisabetta **(F,G)**; gypsum crust at Monte Perrera **(H)**.

Different types of gypsum were investigated from the lower gypsum unit area, including transparent honey-colored selenitic crystals, white crystalline gypsum crystals, and crystalline gypsum aggregates. The samples were obtained from outcrops or fallen blocks (Figure 2). Collected colonized gypsum samples (at least 5 pieces) were sealed in polyethylene bags and stored at room temperature in a dark and dry location until further analysis. The samples were collected in February of 2019. In southern part of Sicily, the long-term average temperature in February is ~13°C, average daylight ~10 h, rainfall ranging from 30 mm to 60 mm and the relative humidity is around 71%.

### 2.2. Micro-CT scan tomography

Six representative samples were chosen to describe the internal textures in detail using X-ray micro-computed tomography (micro-CT scan). The samples were chips or fragments and the dimensions did not exceed 2 cm in their largest dimension.

The micro-CT scan measurements were carried out using a custom-built cone-beam micro-CT scan system, operated in a laboratory of the Institute of Experimental and Applied Physics, Czech Technical University in Prague. The scanner used was equipped with a micro-focus X-ray tube (Hamamatsu L12531), and a CMOS-based flat-panel detector type (Dexela 1512). The tube is characterized by a tungsten transmission target, accelerating voltage up to 110 kVp, 16 W maximal power output, and 2 μm focal spot (considering a 2 W output power). The detector is equipped with a 200 μm thick micro-columnar CsI scintillation crystal and provides an array of 1,944 × 1,536 pixels with a 74.8 μm pixel pitch. Further, the system provides a remote-control multi-axial positioning system, allowing one to accurately set the needed imaging geometry and a precise sample-rotation stage.

The exposition parameters varied based on the actual dimensions of each individual sample. The voxel size of all of the datasets was within the 7.7–13 μm range. The X-ray tube voltage was set to 80 or 90 kVp and was filtered by 2 mm of aluminum and 0.05 mm of copper. The CT scans were carried out with an angle step of 0.15°, resulting in 2400 projections per each dataset. The CT scan reconstruction was performed using filtered back-projection in the dedicated module of VG Studio MAX software (Volume Graphics, Heildelberg, Germany). Data analyses included denoising, ring artifact suppression, segmentation, and subsequent porosity calculation, and was carried out using Dragonfly software (Object Research System, Montreal, Canada). The histogram thresholding and deep learning extension (semantic segmentation models) were used to detect the boundaries of the gypsum fragments in 2D projections and to identify voxels representing empty volumes in the samples. The sum of empty-labeled voxels was used for the determination of the percentage of the apparent porous space in whole fragments or specific zones.

### 2.3. Raman spectroscopy

Selected cells (or colonies) were isolated from the gypsum crystals and put in a drop of water on a microscopic slide. In the case of cyanobacteria, the spectra were collected from these samples using a Renishaw inVia Reflex microspectrometer coupled with a Peltier-cooled CCD detector. Excitation was provided at 514.5 nm and 785 nm (power ~ 0.1–5 mW). Three spectra were recorded in the 100–2,000 cm^−1^ range with a spectral resolution of 1 cm^−1^ full width at half maximum (CCD array detector/CCD chip with 576 × 400 pixels). A single Raman spectrum was collected using 5–10 scans each of 10 s exposure for an improved signal-to-noise ratio. The laser spot size was approx. 1.5 μm in diameter when focused at the surface. By using a 50× objective (NA 0.75), it was possible to analyze single endolithic cells and colonies. A benzonitrile standard was used for spectral wavenumber calibration.

Endolithic coccoid algae from the Monte Gibliscemi site were analyzed using a Thermo Scientific DXR Raman microscope with an Olympus microscope. The spectrometer was calibrated by a software-controlled calibration procedure—using multiple neon emission lines (wavelengths calibration), and multiple polystyrene bands (laser frequency calibration). Spectra were obtained using 532 nm laser (power of 0.1–1 mW, 1,800 line/mm grating). At least 3 spectra were recorded using a high-resolution grating (range of 100–1,860 cm^−1^) with a spectral resolution of 2 cm^−1^. To improve the signal-to-noise ratio, Raman spectra were collected using 64 scans and 2 s exposure. The laser spot size was ~2 μm in diameter when focused on the surface. Endolithic colonies were analyzed using a 50× objective.

All Raman spectra obtained were exported into the Galactic *.SPC format. The spectra were viewed and evaluated using the GRAMS AI spectroscopy software suite (v. 8.0, Thermo Electron Corp., Waltham, MA, United States). The wavenumber positions of the Raman bands were determined using the peak viewing settings and the positions corresponding to the maxima of the peaks.

### 2.4. Fluorescence microscopy

Small pieces of gypsum, colonized by pigmented endoliths were scraped and suspended in double-distilled water. The suspension was stained with SYBR Green I (SBI) (Molecular Probes), which is a fluorochrome specifically used for the staining of nucleic acids. Observations were made first in differential interference contrast (DIC) using a Zeiss AXIO Imager M2 fluorescence microscope (Carl Zeiss, Jena, Germany) plus an Apochrome x60, n = 1.4 Zeiss oil-immersion objective. A CCD Axiocam HRc Rev. 2 camera and AXIOVISION 4.7 software (Carl Zeiss, Oberkochen, Germany) were used to capture and record the DIC images. Images were acquired using a Multichannel Image Acquisition system, employing an eGFP filter set (Zeiss Filter Set 38; Ex/Em: 450–490/500–550 nm), a DAPI filter set (Zeiss Filter Set 49; Ex/Em: 365/420–470 nm), and a Rhodamine filter set (Zeiss Filter Set 20; Ex/Em: 540–552/567–647 nm).

### 2.5. Light microscopy

At least five pieces of colonized gypsum from each site were observed under the light microscope. The biomass (pigmented zones of gypsum) was removed from the stone surface/subsurface area using a sterile laboratory needle, and mechanically crushed on a microscopy slide in a drop of sterile water. If possible, the various pigmented layers were analyzed separately. Samples were observed using an Olympus BX 51 light microscope, equipped with DIC optics, and images of the microalgae were acquired with Olympus DP-72 digital camera using Olympus cell Sens Standard v. 2.1 image analysis software. Cyanobacterial taxa were identified according to Komárek and Anagnostidis (1999, 2005) and Komárek (2013). The relative abundance of individual taxa was estimated and expressed on a semiquantitative scale: dominant species (≥50% of estimated biomass), frequent species (5–49% of the biomass), and scarce species (<5% of the biomass).

### 2.6. SSU rRNA gene barcoding of selected dominant phototrophs

Gypsum samples containing endoliths were selected based on species abundance and overall suitability for the single cell sequencing method (SCS) (e.g., no morphological overlap with other species in the sample) (Table 1).

#### 2.6.1. Cyanobacteria

A modified isolation technique, originally developed for planktonic cyanobacteria, was used, applying a glass capillary under sterile conditions (Zapomělová et al., 2008; Mareš et al., 2015). Colonized areas of the gypsum were scraped off and rehydrated on a microscopy slide in TE buffer (pH = 8), and then mechanically crushed with another microscopy slide. The prepared specimen was observed under the Olympus IX71 inverted microscope and searched for single colonies of selected species. The single colony was separated by repeated washing through a series of droplets of TE buffer, and documented by microphotography. Subsequently, the isolated colony was either directly put into a sterile 0.2 mL Eppendorf tube for further processing (*Nostoc* sp., *Chroococcidiopsis* sp.), or gently crushed using a clean cover slip, with five single cells of the target species collected per tube (*Gloeocapsa* spp., *Gloeocapsopsis pleurocapsoides*). After being kept frozen overnight at −20°C, total genomic DNA from the isolated cells/colonies was amplified using the Multiple Displacement Amplification (MDA) method utilizing Repli-g Mini Kit (Qiagen). The MDA was performed following the manufacturer’s instructions, which involved cell lysis, DNA amplification by Phi 29 polymerase (16 h at 30°C), and a final denaturation step (5 min at 65°C). The quality of MDA products was tested by gel electrophoresis, performed for 75 min at 80 V using 1.5% agarose gel. Successful MDA products were used as a template for PCR amplification of the 16S rRNA gene and the adjacent internal transcribed spacer (ITS) region using the following primer combinations (forwards/reverse): 16S378F/16S1494R, and 16S27F/23S30R (Taton et al., 2003). The quality of the PCR products was tested by gel electrophoresis; performed for 30 min, at 90 V, using 1.5% agarose gel. PCR products were then purified using an Invisorb^®^ Fragment CleanUp Kit (STRATEC Molecular GmbH, Germany) or a Monarch PCR and DNA Cleanup Kit (BioLabs^®^ Inc., New England), and then sent for commercial Sanger sequencing at SeqMe (Dobříš, Czech Republic) using the same primers as for PCR, plus an additional internal sequencing primer (cyano6r) (Mareš et al., 2013).

##### 2.6.1.1. Phylogenetic analysis

A set of SSU (16S) rRNA gene sequences for phylogenetic analysis was compiled from the data obtained in this current study, plus reference sequences from the GenBank database. The final data set included: (i) all sequences collected using the single cell/colony amplification protocol; (ii) the most abundant cyanobacterial amplicon sequence variants (ASVs) from the metagenomic analysis (ASVs reaching a relative abundance of at least 5% in at least one of the analyzed samples) (Benison and Karmanocky, 2014); frequent ASVs highly similar to the sequences of the dominant cyanobacterial morphotypes barcoded using single cell/colony sequencing (ASVs with pairwise sequence identity ≥97% to the single cell/colony sequences and a relative abundance ≥1% in at least one of the analysed samples); and (iv) closely related reference sequences identified by BLASTn against the NCBI non-redundant nucleotide database (using the single cell/colony sequences and abundant ASVs as queries). The resulting set of sequences was aligned using MAFFT v. 7 G-INS-I algorithm (Katoh and Standley, 2013), and subsequent manual removal of those regions with poor coverage. A maximum likelihood tree was reconstructed using RaxML v. 8 (Stamatakis, 2014) with 1,000 rapid bootstrap replications and applying the GTR + G + I substitution model.

##### 2.6.1.2. Metagenomic profiling

For metagenomic analysis, altogether 26 samples from Sites 1 represented by selenitic (1S) and white crystalline-gypsum (1A) and Sites 2 represented by selenitic crystals were selected. Small parts of gypsum crystals exhibiting microbial pigmentation were firstly observed under the light microscope as described above and then scratched off with an ethanol-sterilized scalpel and needle into 2 mL Eppendorf tubes. Prior to DNA extraction, samples were homogenized in Eppendorf tubes using a Retsch MM 200 laboratory mill and 1 mm wolfram carbide beads shaken at 30 s^−1^ frequency for 5 min. Lithobiontic DNA was extracted using the NucleoSpin^®^ Soil Kit (Macherey-Nagel) with lysis buffer SL1 and Enhancer SX, in triplicates per sample and pooled before PCR.

A previously published in-house protocol (Brown et al., 2020) based on Earth Microbiome Project standards (EMP)^1^ was employed for library construction and sequencing. Briefly, samples were amplified and barcoded using fused primers based on 515f and 926r, containing 12-bp Golay barcodes (Parada et al., 2016) and 5-bp barcodes, respectively, in a single PCR reaction (Marotz et al., 2019). The library was cleaned prior to the pooling of PCR products with AMPure XP (Beckman Coulter) magnetic beads; and finally purified using Pippin Prep (Sage Science) to remove non-target DNA, including the eukaryotic 18S rRNA gene PCR product. The library was sequenced on Illumina MiSeq using v3 chemistry with 2 × 300 nt output (Norwegian High Throughput Sequencing Centre and in-house MisSeq platform). The library contained two mock communities as the positive controls, i.e., one gDNA template with an equal composition of 10 bacterial species (ATCC^®^ MSA-1000^™^), and one with a staggered composition of the same bacteria (ATCC^®^ MSA-1001^™^). We have included three negative controls to account for contamination in the PCR library preparation (water template).

We obtained Illumina sequencing data with an average yield of 99654 pairs of reads per sample. Illumina 16S rRNA gene amplicons were initially processed as previously described (Brown et al., 2020) using scripts of USEARCH v. 9.2.64 (Edgar, 2013). The workflow included the initial quality-check (Andrews, 2010), demultiplexing, and primer trimming of the paired reads. The demultiplexed sequencing data with trimmed primers were loaded into R v. 4.1.0 (R Core Team, 2021), run under Rstudio version 1.4.1717 (Rstudio Team, 2021), and analysed following the DADA2 package pipeline v. 1.8 (Callahan et al., 2016) to infer a matrix of ASVs and their abundance in the individual samples. The DADA2 pipeline was run using the default parameters except for the filtering and trimming step, in which the parameters were adjusted to avoid unfavorable effects of any declining quality of the reverse reads by reducing their length [*truncLen = c(210,180)*] and a slight increase in their maximum expected error rate [*maxEE = c(2,5)*]. After the filtering procedures, removal of chimeras, and final merging of paired reads, 32.5% of the best quality reads (in average, 32380 pairs of reads per sample) were retained for further analysis. The hierarchical taxonomic classification of individual ASV was assigned based on SILVA database version 138.1 (Quast et al., 2012). The data set was subsequently imported into the *phyloseq* R package version 1.36.0 (McMurdie and Holmes, 2013), to export the taxonomy and the ASV abundance tables as well as a fasta file with the ASV sequences. The composition of the negative (blank) and positive control samples were checked manually. The control samples were then removed from further statistical analysis, and the data set was decontaminated by deleting minor amounts of contaminant bacteria found in the negative controls (using the *prune_samples* and *prune_taxa* functions in *phyloseq*). Chloroplast, mitochondrial, and eukaryotic sequences were also removed.

To visualize the relative abundance of higher taxa (bacterial phyla, cyanobacterial families) in individual samples using *phyloseq*, the data were normalized at median sequencing depth and rendered as plot bar graphs. To address the variability in sample composition data across defined categories (substrate, colored layer, locality), non-metric multidimensional scaling (NMDS) analysis was performed using R package *vegan* version 2.5.7 (Oksanen, 2009). The data was transformed into Bray-Curtis distances, and the statistical significance of differences between the categories was tested using permutational anova function *adonis* (999 permutations). Differences in alpha-diversity across the defined categories of samples were tested using pairwise t-tests (R package *stats* version) of diversity indices (Shannon, Simpson) calculated using the *diversity* function. The differences in the abundance of individual taxa across the defined categories of samples were tested using pairwise t-tests, and abundance data on individual taxa available as a *phyloseq* output.

#### 2.6.2. Algae

Coccoid green algae were mostly present in orange subsurface zones of some gypsum samples. Endolithic algae were isolated from crystalline gypsum rock from Monte Gibliscemi (Site 4). The crystalline gypsum from this site was selected due to the highly abundant and easily recognized orange-pigmented algal cells, without the presence of any other autotrophic species.

DNA was extracted from the orange colonization using the commercial kit DNeasy^®^ PowerLyzer^®^ PowerSoil^®^ Kit (Qiagen) as described in the provided protocol. After being briefly vortexed again, the samples were centrifuged at 12,000 g for 2 min, the supernatant was used for testing DNA quality (isolate S14 Sicily) on NanoDrop^®^ND–1000 Spectrophotometer (NanoDrop Technologies, Inc.). The 18S rDNA region was then amplified by PCR using the forward primer FC (GGGAGGTAGTGACAAIAAATA), and the reverse primer RF (CCCGTGTTGAGTCAAATTAAG) (Matsuzaki et al., 2015). Each 21.44 μL of PCR reaction for 18S rDNA amplification contained 2 μL of DNA isolates (diluted to a concentration of 5 ng.μL^–1^), 4.32 μL 5x MyTaq Red Reaction Buffer (Bioline, Meridian Bioscience, United States), 0.43 μL of each 10 μM primer, 14.04 μL sterile Milli-Q water, and 0.22 μL of 5 U.μL^−1^ MyTaq HS Red DNA polymerase (Bioline, Meridian Bioscience, United States). Amplification reactions were performed using the following cycle parameters: for 3 min initial denaturation at 95°C, followed by 35 cycles (denaturation for 15 s at 95°C, annealing for 30 s at 56°C, extension for 40 s at 72°C), and final extension for 7 min at 72°C. The quality of the PCR products was tested by gel electrophoresis (45 min, 120 V, 1.5% agarose gel). PCR products were purified and sequenced using an Applied Biosystems automated sequencer (ABI 3730xl) at Macrogen Europe (Amsterdam, Netherlands). Chromatograms of the obtained sequences were examined using the FinchTV 1.4.0 (Geospiza, USA) program. Only sequences showing distinct single peaks in the electropherogram were used.

18S rDNA alignments were constructed for the phylogenetic analyses. The sequences were selected according to Gálvez et al. (2021) to encompass the closest relatives, to isolate S14 Sicily and other lineages in Chlamydomonadales. The 18S rDNA alignment contained 56 sequences (1,682 bp); the representatives of clade *Polytominia* and *Dunaliellinia* were selected as the outgroup. The best-fit nucleotide substitution model was estimated by jModeltest 2.0.1(Posada, 2008). Based on the Akaike information criterion, the GTR + I + G model was selected for 18S rDNA. The phylogenetic trees were inferred by Bayesian inference (BI) and maximum likelihood (ML) according to Nedbalová et al. (2017), with a minor modification that the Markov chain Monte Carlo runs were carried out for 3 million generations in BI. Bootstrap analyses and Bayesian posterior probabilities were performed as described by Nedbalová et al. (2017).

All obtained sequences were compared to those in GenBank by BLAST and were submitted to the NIH genetic sequence database GenBank at NCBI during paper revision under accession numbers OQ509475 − OQ509403 and OQ520113.

## 3. Results

In this study, samples collected from four different sites in Sicily were initially analyzed by light microscopy. From these samples, the most abundant phototrophic endoliths were then sequenced and analysed using Raman spectroscopy. Based on the availability of samples with optimal biomass (i.e., at least 10–20 mg of dry mass) levels and different gypsum varieties present, two sites (Site 1 and 2) have been selected for metagenomic analysis. This allowed us to gain insights into the microbial diversity and functional capabilities of the samples.

### 3.1. Visual observation of colonized gypsum

In the field, gypsum rocks from 4 sites in Southern Sicily were evaluated for lithic colonization by visual examination of various coloration in fractured samples. The presence of colonization were also confirmed on the outcrops by the detection of biological pigments such as carotenoids by portable Raman spectrometer as previously described (Němečková et al., 2020).

At the **Siculiana Marina (Site 1)** two gypsum varieties were sampled—selenitic (1S) and white crystalline-gypsum (1A). Flat, thin, transparent selenitic crystal aggregates (each crystal was about 5–10 cm long and 3–5 cm wide) were found at Site 1 (Figure 2D). Aggregates were oriented in various directions on the outcrop. The inner parts of the crystals were slightly weathered, and after removing the crystals from the outcrop, a coarse-grained weathered matrix was revealed. Black and green pigmented endoliths were mainly observed in cleavage spaces of selenites and occasionally in coarse-grained matrix. The black-pigmented colonizations were always found in the uppermost areas closer to the sun-exposed zones while green colonizations were found below them. At the same outcrop, tabular white crystal aggregates were also observed (Figure 2E). Aggregates were around 10 cm long and wide, and around 5 cm thick. On the outcrop, aggregates were unordered and weathered in their inner parts, forming a fine-grained matrix beneath. The colonization had the same features as the colonization of selenitic crystals, i.e., the uppermost dark pigmented endoliths and inner green colonizations.

**Santa Elisabetta (Site 2)** contained flat, thin, transparent selenitic crystals (Figures 2F,G). Crystals were about 15–20 cm long and 5–10 cm wide. The character of the endolithic colonization was similar as in the case of 1S Site, but additional scarce orange zones were present between black and green colonizations.

**Monte Perrera (Site 3)** was characterized by a white and hard gypsum crust on south-east side of Monte Perrera Mountain (Figure 2H). In some areas, cracks colonized by dark endoliths were observed. Below the crust, endolithic colonization zones were in the fine-grained matrix underneath.

**Figure 3:**
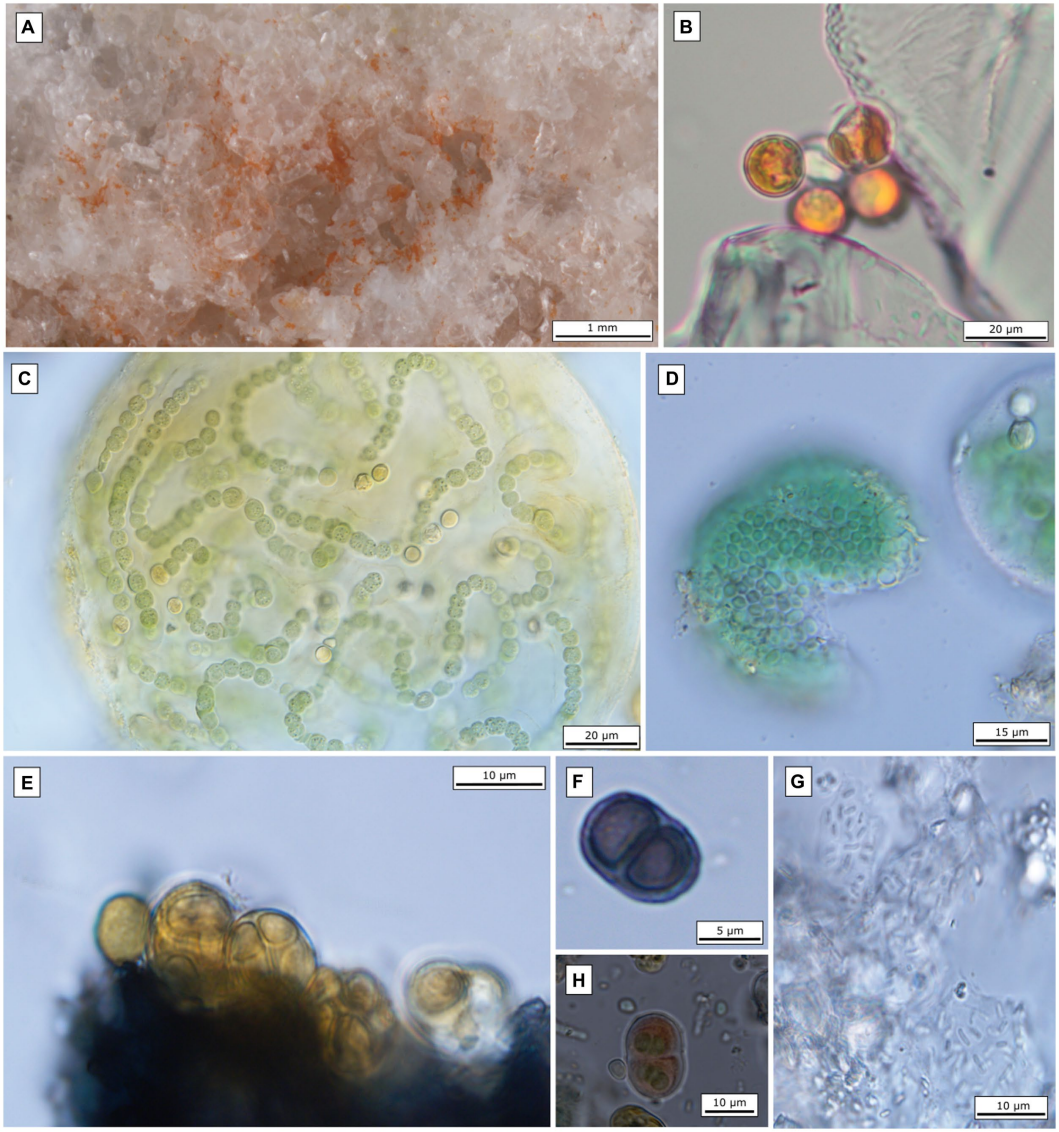
Microscopic images of the most abundant endolithic algae and cyanobacteria. **(A)** Gypsum crystals colonized by orange-pigmented algae at site 4; **(B)** microscopic image of coccoid algae isolated from the field gypsum sample (site 4); **(C)** *Nostoc* sp. (isolated from site Siculiana Marina); **(D)** *Chroococcidiopsis* sp. (isolated from site Siculiana Marina); **(E)** *Gloeocapsopsis pleurocapsoides* (isolated from site Monte Perrera); **(F)** *Gloeocapsa compacta* (isolated from site Monte Perrera); **(G)** *Gloeobacter violaceus* (isolated from site Monte Perrera); **(H)** *Gloeocapsa novacekii* (isolated from site Santa Elisabetta).

**Monte Gibliscemi (Site 4)** contained fallen blocks close to the western side of the mountain Monte Gibliscemi that were colonized by orange-pigmented endoliths found in the upper parts of fine-grained gypsum (Figure 3A).

### 3.2. Micro-CT scans characterization of gypsum

To evaluate some of the textural features of selected specimens of gypsum, micro-CT scan analyses were conducted. Image analysis allowed us to investigate the three-dimensional inner architecture of gypsum fragments. A basic description of the observed characteristics and parameters is summarized in Table 2. Additional graphic materials of the mapped void space can be found in Supplementary material.

**Table 2:** Relative abundace of cyanobacteria obtained by light microscopy.

Site	Layer	Overall relative abundance of observed phototrophs		
		< 5%	5–49%	> 50%
SM (1S)	Black	*Gloeobacter violaceus*, *Nostoc* sp., *Chroococcidiopsis* sp., *G. novacekii, Petalonema* sp., *Anathece* sp.	*Chroococcidiopsis* sp., *G. rupestris*, *Gloeocapsa* sp., Gloeocapsopsis pleurocapsoides*, *Chlorophyta*	*Nostoc* sp.*., *G. compacta*, *Petalonema* sp.
	Green	*Nostoc* sp., *Microcoleus* sp., *Anathece* sp., *Gloeocapsa novacekii*, *G. rupestris*, *G. compacta*, *Chroococcus* sp.	*Chroococcidiopsis* sp., *Chroococus* sp., *Gloeocapsa* sp., *G. rupestris*, *G. novacekii*, *Chlorophyta*	*Chroococcidiopsis* sp.
SM (1A)	Black	*Petalonema* sp., *Chroococcidiopsis* sp., *Nostoc* sp., *G. novacekii*, *Gloeocapsa*, sp.	*G. compacta*, *G. biformis*, *Gloeobacter violaceus*, *Gloeocapsa* sp., *G. rupestris, Chroococcidiopsis* sp., *Petalonema* sp., *G. pleurocapsoides*	
	Green	*G. compacta, Gloeocapsa* sp., *Nostoc* sp.	*Nostoc* sp.	*Chroococcidiopsis* sp.
SE (2)	Black	*Chroococcidiopsis*, *Chlorophyta*	*Gloeocapsa* sp., *Nostoc* sp., *G. biformis*, *G. rupestris*, G.novacekii*, *Chlorophyta*	*Gloeobacter violaceus*, *Chroococcidiopsis* sp.
	Green	*Petalonema* sp., *Chroococcidiopsis* sp.	*Chroococcidiopsis* sp., *Nostoc* sp., *Chlorophyta*	*Chroococcidiopsis* sp., *Gloeocapsa* sp., *Gloeobacter violaceus*
MP (3)	Black	*Nostoc* sp.	*G. pleurocapsoides*	Gloeocapsa compacta*
	Orange	*Gloeobacter violaceus*		
	Green	*G. pleurocapsoides*	*Chroococcidiopsis* sp.	
MG (4)	Orange	Chlorophyta*		

*phototrophs selected for single cell sequencing.

**Figure 4:**
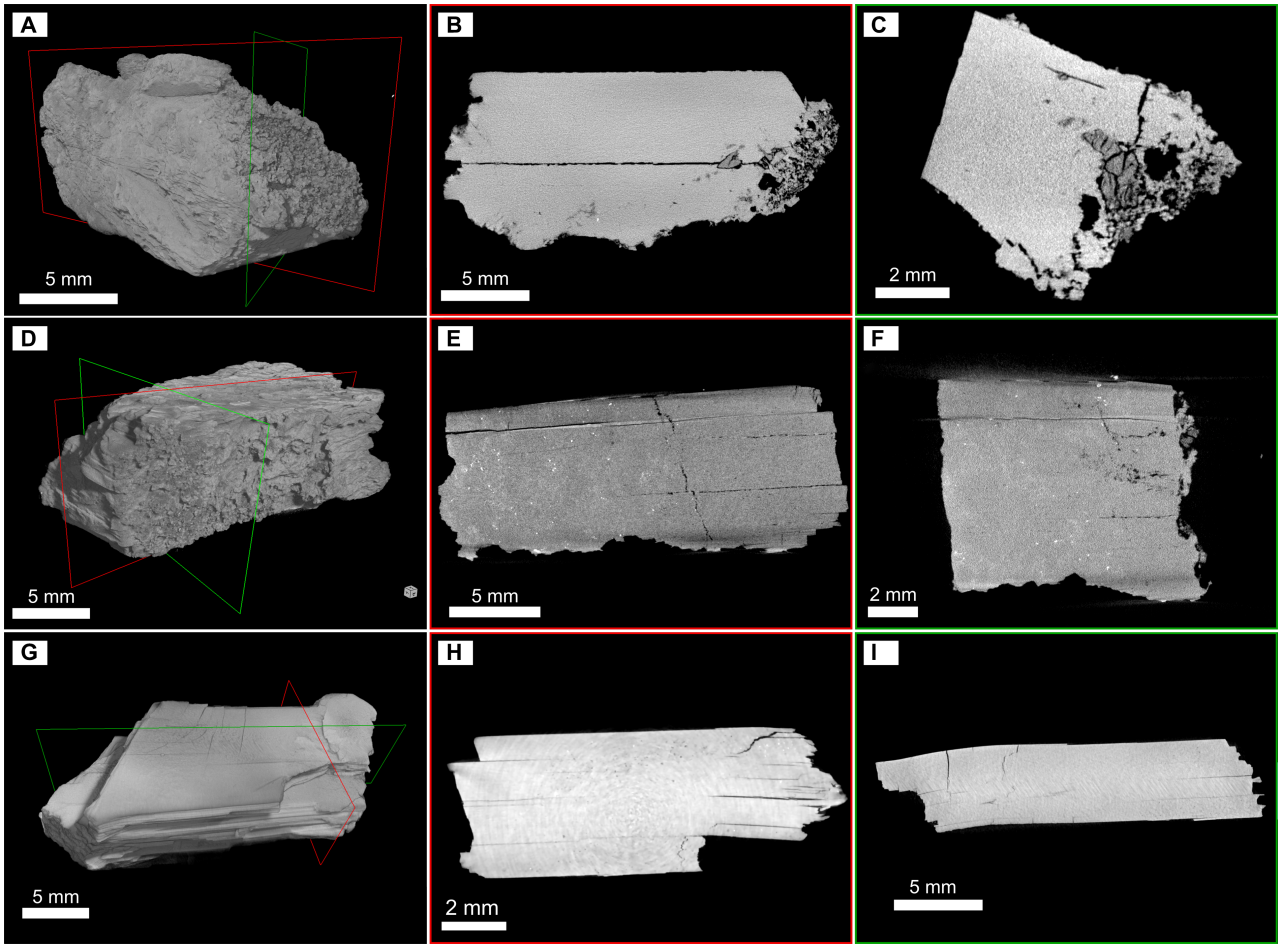
Micro-CT 3D reconstructions and selected slices illustrating the inner architecture and void space of gypsum fragments. Samples from Siculiana Marina—1A **(A–C)** and 1S **(D–F)**; and from Santa Elisabetta **(G–I)**. The red and green frames in the 3D images show the position of the displayed slices.

**Siculiana Marina (Site 1, 1S, 1A):** Gypsum from the Siculiana Marina site was composed of dense tabular crystals surrounded by secondary rims or crusts, which was observed for two gypsum samples (1A and 1S, Figures 4A,D). The crystals were predominantly broken by several cracks along the main cleavage plane, locally disturbed by cracks of different orientation (Figures 4B,C,E,F). These mostly linear features are generally open at the margins. Another system of small cracks and cavities that ignored the cleavage was also present, but these were predominantly isolated in the matrix, narrow, and not easily accessible from the surface. The crystal surfaces were corroded, and shallow etch pits were visible mainly along the cleavage plane. However, in the case of the 1S sample, most of the corrosion marks are covered by secondary crusts. The calculated void space, including large cracks, was approximately 1.2 and 0.8% for the 1A and 1S samples. In contrast, the secondary crusts of the samples showed an entirely different, spongy-like texture that forms an open and highly interconnected porous space. The 1A sample was characteristic for the well-preserved secondary crust (Figures 4B,C), where the pores occupied approximately 15.1% of the volume. The remaining secondary rim on the 1S sample was less than 1 mm thick but contained a similar porous space of 17.5%.

**Santa Elisabetta (Site 2, SE):** Micro-CT scan reconstruction of the SE sample revealed tabular well-developed crystals (Figure 4G) corresponding to the selenitic nature of gypsum found on this site. The investigated fragment was unweathered, but several linear fissures along the main cleavage plane were separating the individual flat crystals (Figures 4H,I). Occasionally, cracks of a different orientation were observed. This gypsum was predominantly dense, except for some domains where narrow cracks within the matrix could be observed. The void space occupied only 1.8% of the total volume of the fragment.

**Monte Perrera (Site 3, MP):** According to the micro-CT scan images, gypsum from the Monte Perrera site was represented by aggregates of oriented subhedral tabular-prismatic coarse crystals, which also were partially affected by corrosion, dissolution, and recrystallization. Micro-CT scan reconstruction of the MP1 sample showed that the gypsum grains are tightly packed (Supplementary Figure S1A), and three zones can be distinguished in the fragment (Supplementary Figures S1B,C). The first zone consisted of large gypsum grains with relatively narrow and tiny fissures, but with relatively large cavities. The second zone was represented by smaller elongated gypsum crystals, but, in contrast, they were frequently separated by more open fissures coupled with cavities, which were probably formed by dissolution of the gypsum. Together, the interstices formed approximately 14.8% of the void space in the crystalline part of the aggregate. The preserved remains of the secondary recrystallized gypsum crust represented the third zone. It was partially fractured with a network of small cracks, but it also contained large spheroidal cavities. These cavities are responsible for a relatively large volume of empty space in the crust (23.4%), although the cracks in the matrix are smaller than in the crystalline portion. The CT scan images of the MP2 sample illustrated how the crystalline core was surrounded by the secondary gypsum crust (Supplementary Figure S1D). Individual subhedral crystals can still be observed in the core, but the shape of the grains and the interstices between them may suggest that the gypsum grains were deeply corroded. There was no distinct boundary, and the gypsum grains gradually transitioned into a fragmented and probably recrystallized mass, which also contained a system of small cracks and larger cavities (Figures 4E,F). The calculation performed for the part of the sample, where the crystalline core was intentionally excluded, showed that the crust matrix was relatively dense with approximately 16.4% of void space.

**Monte Gibliscemi (Site 4, MG):** The MG sample was composed of coarse subhedral tabular-prismatic crystals that were cemented by a crystalline matrix (Supplementary Figure S1G). The CT scans revealed fissures of different sizes between large gypsum crystals, and, in some cases, cracks along the cleavage plane could be found. A similar texture was also observed for the matrix, although on a different scale with smaller gypsum grains and subtle fissures (Supplementary Figures S1H,I). In general, this fragment is highly compacted, with interstices occupying 8.1% of the sample volume.

### 3.3. Bright field microscopy of endolithic phototrophs

**At Siculiana Marina (Site 1)** and **Santa Elisabetta (Site 2)**, black pigmented colonies were composed predominantly of *Nostoc* sp. and/or *Gloeocapsa compacta* in those zones closer to the surface (distanced around 0.5–1 cm from the surface), while green colonization dominated by *Chroococcidiopsis* sp. was located underneath (Figure 3D). The black pigmented colonizations were more diverse, contained predominantly *G. compacta* and *Nostoc* sp. (but also other species—*G. biformis, G. novacekii, Chrococcidiopsis* sp. and *Gloeobacter violaceus*) (Figure 3). These black zones transformed diffusely into the inner green pigmented layer dominated by *Chroococcidiopsis* species.

**Monte Perrera site (Site 3)** showed a less diverse endolithic community. The uppermost black layer (around 1 cm from the surface) dominated by *G. compacta* was present. Below that, sporadic orange colonization of *G. violaceus* only, and inner green zones of predominated by *Chroococcidiopsis* sp. colonizations were recognised.

At **Monte Gibliscemi (Site 4)**, gypsum rocks were colonized by orange-pigmented coccoid cells of *Chlorophyta*, which were found in the upper parts of fine-grained gypsum without the visible presence of other endolithic organisms. The colonizations were around 0.5–4 cm from the surface (Figure 3B).

### 3.4. Raman microanalysis of endolithic pigments

**Figure 5:**
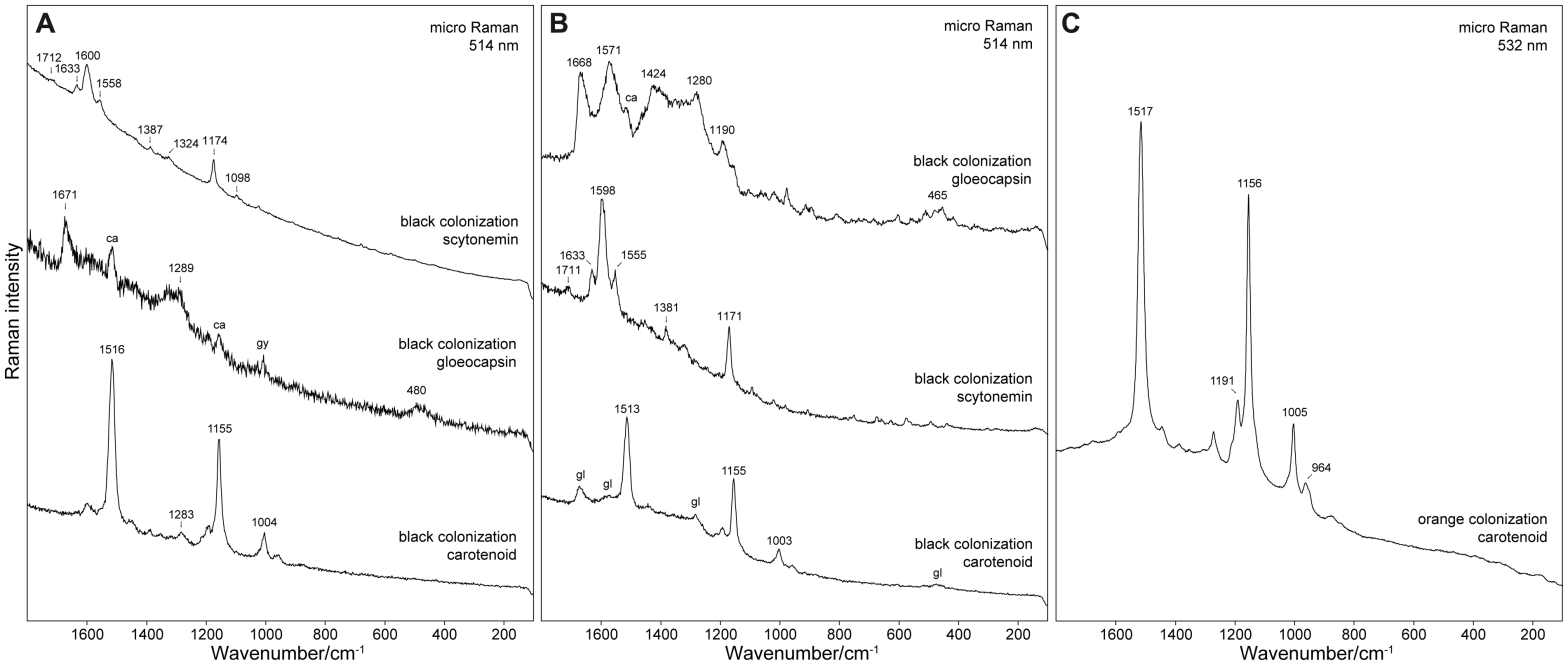
Raman spectra of pigments detected in samples from **(A)** Siculiana Marina, **(B)** Monte Perrera, and **(C)** Monte Gibliscemi sites. Abbreviations: ca, carotenoid; gl, gloeocapsin; gy, gypsum.

Raman spectroscopy allowed the detecting of the dominant pigments *in situ* in selected parts of the investigated specimens. In those samples in the uppermost dark pigmented layer, specialized pigments that protect against increased UV-radiation were detected. As mentioned above, these upper layers were colonized mostly by *Nostoc* sp., *Gloeocapsa* sp. (especially *G. compacta*), and *Chroococcidiopsis* sp. Raman bands of detected pigments and their Raman spectra are shown in Figure 5, respectively.

Besides scytonemin (major pigment of *Nostoc* sp.) and gloeocapsin (major pigment of *Gloeocapsa* sp.), carotenoids were also detected in all analysed cell or colonies. As a matter of fact, it is very common that the Raman spectra of even the single cells contain the signal of carotenoids together with the signal of other pigments. This can be explained by the substantially increased sensitivity for the carotenoids due to resonance enhancement. The presented spectra of scytonemin and gloeocapsin in Figure 5 were selected as examples where the contribution of the Raman signal of carotenoids was minimal. Carotenoids were also detected in the green pigmented zones below. Carotenoids were the only protective pigments detected in the orange algae collected from Site 4. Raman bands of detected pigments can be found in Supplementary Table S1.

#### 3.4.1. Siculiana Marina (site 1)

At this site the major organism identified in the black layer was *Nostoc* sp., with the dominant pigment scytonemin. Detected Raman bands of scytonemin were 1,712 (weak intensity), 1,633 (medium), 1,600 (strong), 1,558 (m), 1,387 (w), 1,324 (w), 1,174 (ms), 1,098 (mw), 1,023 (w), 755 (w), and 681 (w) cm^−1^. The most significant Raman band of scytonemin was detected at 1,600 cm^−1^, corresponding to the ν(CCH) aromatic ring quadrant stretching mode. The band at 1,558 can be assigned to the ν(CCH) p-disubstituted aromatic ring, ν(CCH) p-disubstituted aromatic ring, and the characteristic band at 1,174 cm^−1^ of the ν(C=C–C=C) system (Edwards et al., 2000).

*Gloeocapsa compacta* was another major organism in the black layer with the dominant pigment gloeocapsin. Its Raman bands were detected at 1,671 (m), 1,289 (m), and 480 (mw) cm^−1^. The analysis of *Gloeocapsopsis pleurocapsoides* cells revealed a strong carotenoid signal at 1,516 (*vs*), 1,449 (w), 1,388 (w), 1,283 (mw), 1,189 (mw), 1,155 (ms), 1,004 (m), and 957 (w) cm^−1^. The band at 1,516 cm^−1^ represents the C=C stretching mode of the conjugated chain in carotenoids. The band at 1,155 cm^−1^ band represents the C–C stretching mode (coupled with C–H in-plane bending); and the band at 1,004 cm^−1^ band is attributed to the C–CH_3_ rocking mode.

#### 3.4.2. Monte Perrera site (site 3)

*Gloeocapsa compacta* was the dominant species in the black layer, and the pigment gloeocapsin was detected as the major pigment of this cyanobacteria. The detected Raman bands were at 1,668 (s), 1,571 (s), 1,424 (ms), 1,340 (m), 1,280 (ms), 1,190 (m), and 465 (m) cm^−1^. The bands around 1,668 cm^−1^ can be assigned to *ν*(C=O) vibration, the bands around 1,571 cm^−1^ to *ν*(C=C) aromatic ring vibration, and the bands around 1,276 cm^−1^ to δ(CH_2_) vibration. The signal of carotenoid pigments was also detected in *G. compacta*, as corroborated by its Raman bands at: 1,513 (vs), 1,283 (w), 1,193 (m), 1,155 (s), 1,003 (m), and 958 (w). The pigment scytonemin was detected as a major pigment in *Nostoc* sp. cells: 1,711 (w), 1,633 (m), 1,598 (s), 1,555 (m), 1,381 (mw), 1,171 (ms), 573 (w), and 436 (w) cm^−1^ (see Figure 5B).

#### 3.4.3. Monte Gibliscemi (site 4)

At this site the orange layer was colonized by orange-pigmented coccoid cells of *Chlorophyta* as corroborated by the strong signal of carotenoid pigment that was detected with bands at 1,517 (s), 1,447 (w), 1,389 (w), 1,274 (w), 1,191 (ms), 1,156 (s), 1,005 (m), and 964 (w) cm^−1^ (see Figure 5C).

### 3.5. Fluorescence microscopy of endolithic cyanobacteria

**Figure 6:**
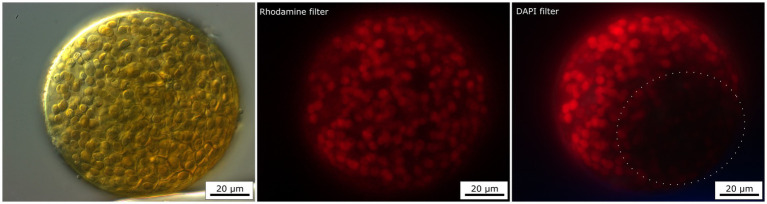
Fluorescence image of *Nostoc* colony using two different filters—Rhodamine and DAPI filter. The red autofluorescence of chlorophyll *a* and phycobiliproteins are partly screened by the presence of brown UV-screening pigment—scytonemin when excited by DAPI filter (dotted circle).

The presence of UV-screening pigment such as scytonemin within some cyanobacteria aggregates were indirectly detected by the visualization of autofluorescence of phycobiliproteins, and chlorophyll *a* signal attenuation observed using a DAPI filter but not using a Rhodamine filter. The excitation light of the DAPI filter (maximum at 365 nm) lay in UVA radiation range. Indeed, this UVA radiation can be absorbed by the UV-screening scytonemin, if present within the cyanobacteria aggregates. This situation is shown in Figure 6, where a large colony of *Nostoc* species in dark-pigmented part (DIC image) of the aggregate revealed a distinct attenuation of the autofluorescence signal when visualized with a DAPI filter. The same cyanobacteria aggregate had exhibited a uniform autofluorescence signal over the aggregate when observed with the Rhodamine filter, as scytonemin does not absorb light in the Rhodamine filter excitation range (450–490 nm). Similar results were also observed for other cyanobacteria taxa such as *G. pleurocapsoides* and *G. compacta*.

### 3.6. Molecular analysis

We used molecular methods based on rRNA for the phylogenetic barcoding of the most abundant species found—coccoid algae, *G. compacta*, *G. novacekii*, *G. pleurocapsoides*, *Nostoc* sp., and *Chroococcidiopsis* sp., as well as for the overall metagenomic studies of entire communities across two different varieties of gypsum (selenite vs. crystalline gypsum) and two differently macroscopically pigmented layers of colonizations (green vs. black) (Supplementary Table S2).

#### 3.6.1. Metagenomic profiling of cyanobacteria

**Figure 7:**
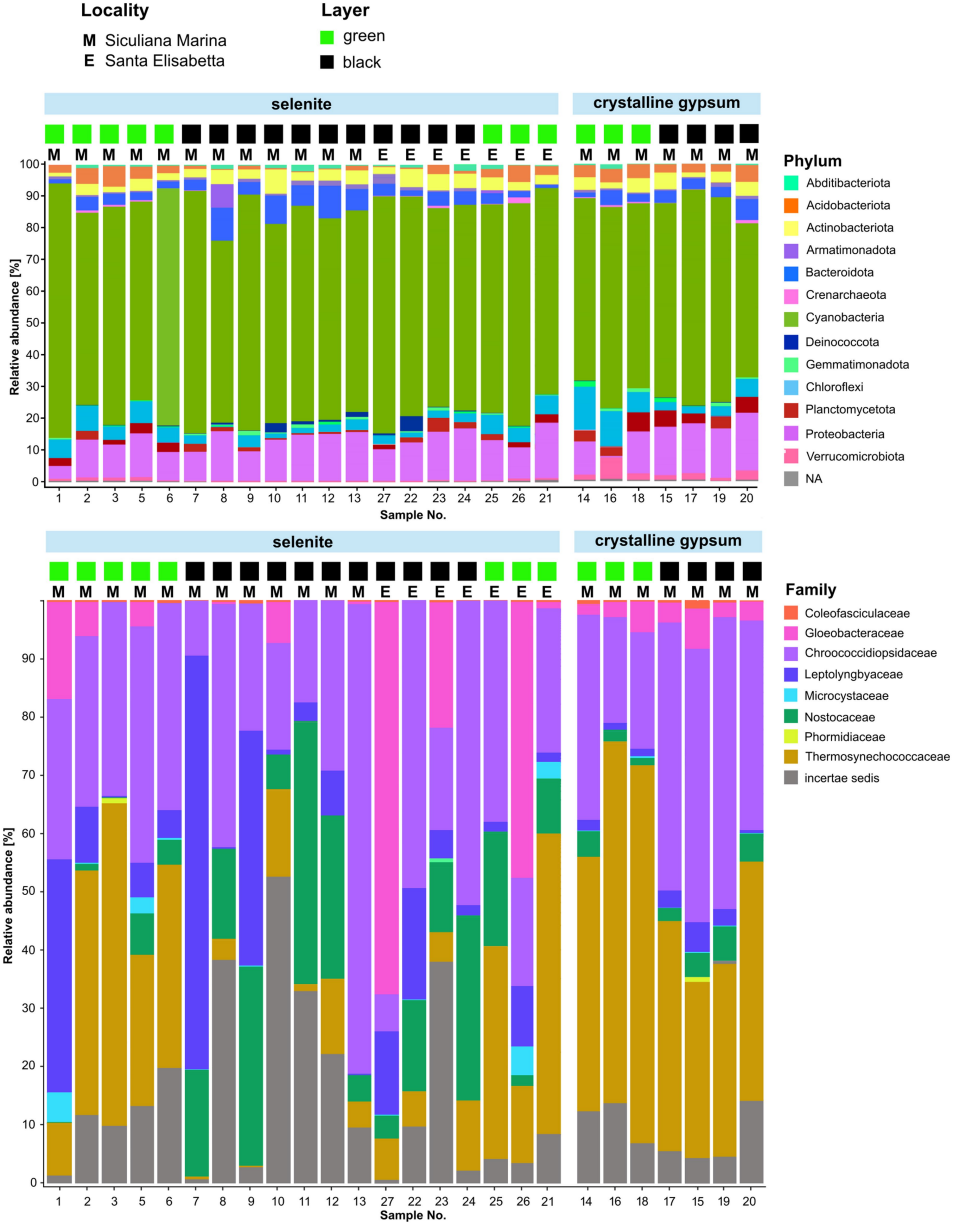
Graphical representation of the relative abundance of prokaryotic phyla and cyanobacterial families in two locations (Siculiana Marina and Santa Elisabetta), in selenitic and crystalline gypsum and in differently pigmented endolithic zones (green versus black).

We obtained a total of 5,424 unique bacterial ASVs of the 16S rRNA gene, with 100–805 (354 on average) ASVs per sample. Taxonomic assignment of the ASVs to bacterial phyla consistently showed that the dominant group was Cyanobacteria (65.9% on average), followed by Proteobacteria (12.6%), Chloroflexi (4.5%), Bacteroidota (4.2%), Actinobacteriota (3.6%), Acidobacteriota (2.5%), Planctomycetota (2.3%), and Verrucomicrobiota (0.9%)—the latter showing a higher abundance in crystalline gypsum samples (Figure 7). Two families of coccoid cyanobacteria were the most abundant across the majority of the samples: Chroococcidiopsidaceae and Thermosynechococcaceae (Figure 7). Members of Leptolyngbyaceae, Nostocaceae, and Gloeobacteraceae were also generally present, and each of those groups was dominant in several individual samples.

Of the two localities for which sequencing data were obtained, Sicualiana Marina contained both types of the lithic substrate; whereas Santa Elisabetta contained only the selenite substrate. However, both localities included sets of green and black colored endolithic colonizations. Therefore, the data set was only partially representative with regard to the two types of gypsum; and the effect of the substrate type could only partly be separated from any unknown effects of the sampling locality such as, for example, the differences in the nutrient availability. Nevertheless, when we looked at the taxonomic composition between the two localities, the only striking difference was the dominance of Gloeobacteraceae in two (black and green) analyzed selenite samples collected from the Santa Elisabetta site. The two localities did not exhibit a significant difference in average biodiversity (Shannon index; *p* > 0.1). The samples from Santa Elisabetta were more uniform (Supplementary Figure S1), which was not surprising given their lower number and occurrence of only selenite.

In terms of the type of substrate, selenites on average contained more Nostocaceae, Leptolyngbyaceae, and Gloeobacteraceae (*p* < 0.05); but less Thermosynechococcaceae (*p* < 0.01) when compared to crystalline gypsum (Figure 7). The composition of the crystalline gypsum samples was more uniform, and all of them were only dominated by Chroococcidiopsidaceae and Thermosynechococcaceae. However, in the crystalline gypsum samples, Gloeobacteraceae and Coleofasciculaceae sequences were always present, while missing in some samples of the selenites. In summary, these patterns could be expressed as a significantly higher average biodiversity (Shannon index; *p* < 0.01), but a lower overall variability (Supplementary Figure S2) in crystalline gypsum versus selenite samples.

The dark-pigmented endolithic zones on average contained more Nostocaceae (filamentous cyanobacteria with colored sheaths) compared to the green-pigmented zones (*p* < 0.05) (Figure 7). On the other hand, Thermosynechococcaceae (coccoid cyanobacteria with colorless envelopes) were more abundant in the green areas (*p* < 0.01). Chroococcidiopsidaceae (coccoid cyanobacteria with pigmented or non-pigmented envelopes) were present in high abundance within both of these differently colored zones. The biodiversity was not statistically different between the black-pigmented and green zones (Shannon index; *p* >0.1) however, the samples from green endolithic zones were more homogenous (Supplementary Figure S3).

Significant differences in the overall ASV composition among the endolithic assemblages from selenite vs. white crystalline-gypsum, samples from green vs. dark layers, and samples from the two localities with sequencing data were further supported by NMDS analysis (Supplementary Figures S1–S3); although the variance between the localities was less pronounced. In permutational ANOVA tests, the type of substrate, the color of the endolithic layer, and the locality significantly explained 12% (*p* < 0.001), 10% (*p* < 0.001), and 9% (*p* < 0.01) of the observed variability, respectively.

#### 3.6.2. Phylogenetic analysis

Phylogenetic analysis allowed us to link the majority of the dominant ASVs discovered by metagenome profiling to cyanobacterial morphotypes observed by light microscopy.

**Figure 8:**
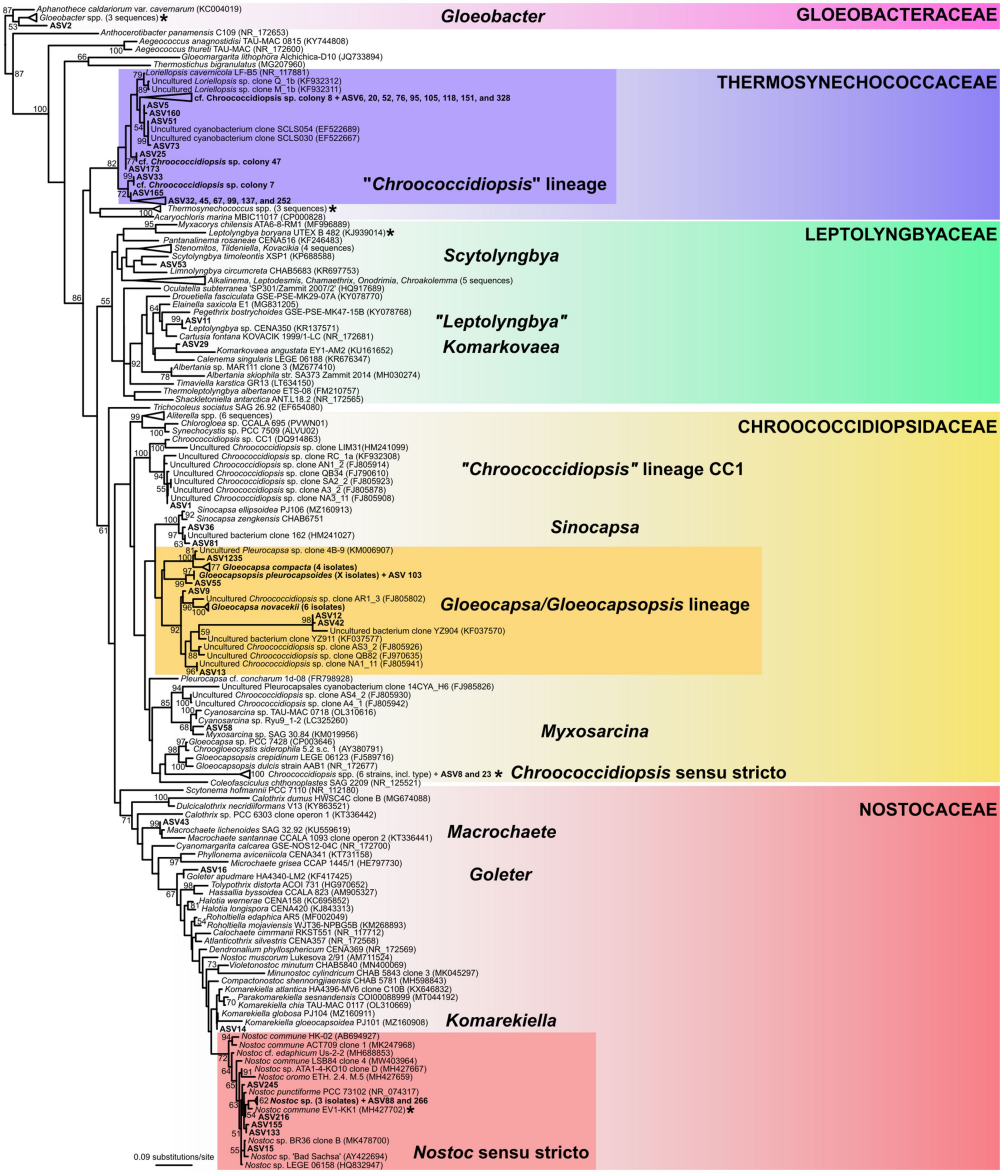
16S rRNA gene phylogenetic tree of cyanobacteria. The position of cyanobacterial morphotypes from the gypsum samples, barcoded by single cell sequencing, is shown in the tree along with the most abundant amplicon sequence variants (ASV) from metagenome analysis. Position of cyanobacterial families and genus-level lineages that were dominant in the metagenome analysis is highlighted. Position of the type species of the families is indicated by asterisks. The tree was inferred using Maximum Likelihood algorithm with 1,000 bootstrap pseudo-replicates, branch supports >50% are shown at nodes.

The largest subgroup of dominant ASVs were classified to Thermosynechococcaceae (see Figure 8 for the list of ASVs), clustering in a deep-branching monophyletic lineage together with three of our single cell isolates of the “*Chroococcidiopsis*” morphotype. Another dominant group of ASVs, classified as Chroococcidiopsidaceae, exhibited considerable taxonomic diversity. It contained ASVs that could be assigned to several species within the lineage of *Gloeocapsa* spp. and *Gloeocapsopsis pleurocapsoides* (ASV9, 12, 13, 42, 55, 103, and 1,235), as established by our single cell isolates. Furthermore, it contained two abundant ASVs (8 and 23) clustering in the *Chroococcidiopsis sensu stricto* clade defined by the sequence of its type species, *C. thermalis*, two ASVs (36 and 81) in the *Sinocapsa* lineage, one very abundant “*Chroococcidiopsis* sp.” of the CC1 lineage (ASV1), and a single ASV (58) related to *Myxosarcina*. These results demonstrate extensive cryptic diversity of the coccoid endolithic cyanobacteria in the samples, especially regarding the *Chroococcidiopsis*-like morphotype.

Another very abundant ASV (2), classified as Gloeobacteraceae, was resolved as a sister lineage to *Gloeobacter violaceus*, in agreement with the observation of *G. violaceus* by light microscopy. The three most abundant ASVs of Leptolyngbyaceae were resolved in clades corresponding to *Scytolyngbya* (ASV53), *Komarkovaea* (ASV29), and “*Leptolyngbya*” sp. (ASV11); outside of the core *Leptolyngbya* cluster, showing the relatively broad diversity of simple filamentous cyanobacteria in the samples. These taxa were not captured by microscopic analysis (Table 2). A majority of ASVs classified as Nostocaceae were resolved in the *Nostoc sensu stricto* clade along with our sequences of *Nostoc* sp. single filaments identified by light microscopy. The remaining three abundant ASVs of heterocytous cyanobacteria clustered into genera *Goleter* (ASV16) and *Komarekiella* (ASV14), which are morphologically cryptic to *Nostoc*, and to *Macrochaete* (ASV43), a heteropolar cyanobacterium which was not found by microscopy.

Several taxa identified in the samples using light microscopy, i.e., *Petalonema* sp., *Microcoleus* sp., and *Anathece* sp., were not found among the dominant ASVs; however, sequences classified as *Petalonema* and *Microcoleus* were found in the samples as low-abundance ASVs.

#### 3.6.3. Sanger sequencing of orange-pigmented coccoid algae and their phylogenetic position

Using culture-independent sequencing, the acquired Sanger sequence (385 bp long) matched with 98.96% identity to several closely related strains or environmental sequences that were all assigned to the order Chlamydomonadales (Chlorophyta), clade *Stephanosphaerinia*: “*Chloromonas*” sp. KSF063 (MH400034.1), “*Chloromonas*” sp. KNF032 (MH400032.1), “*Chloromonas*” sp. KNF030 (MH400031.1), “*Chloromonas*” sp. 435258601_3_18SR_D09 (MK330880.1), “*Chloromonas*” sp. 435258601_3_18SF_F09 (MK330879.1), uncultured alga gene Otu011 (LC371433.1), “*Chloromonas*” sp. KNF0032 (KU886306.1), *Chlorominima collina* CCAP 6/1 (MW553075), and *Chlorominima collina* CCCryo 273–06 (HQ404890.1).

**Figure 9:**
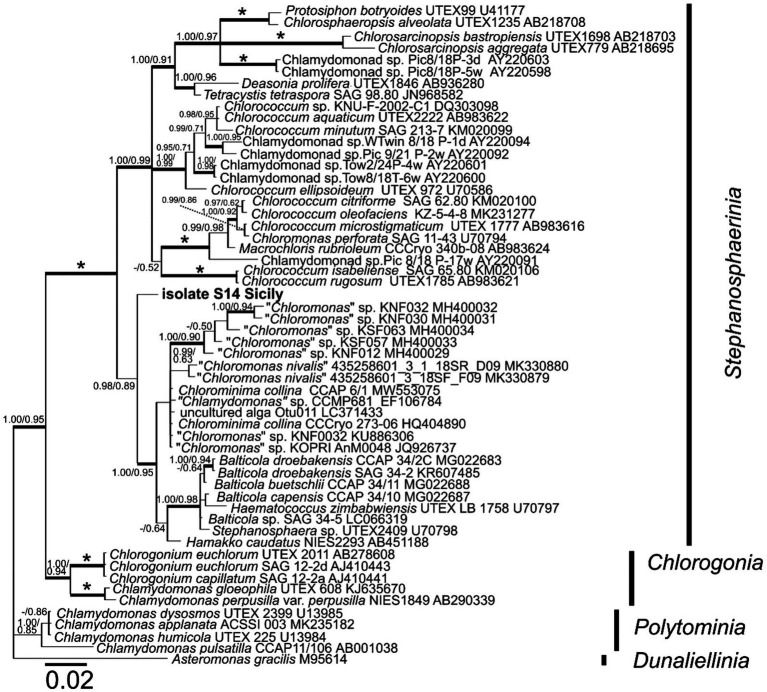
18S ribosomal RNA gene-based Bayesian phylogenetic tree including isolate S14 Sicily (in bold, accession number OQ520113) and other related Chlamydomonadales. Bootstrap values from maximum likelihood analysis (≥50%) and posterior probabilities (≥0.95) are shown. Full statistical support (1.00/100) is marked with an asterisk. Thick branches represent nodes receiving the highest posterior probability support (1.00). Accession number, strain, or field sample codes are indicated after each species name.

Phylogenetic analyses of the 18S rDNA marker placed isolate S14 Sicily in the clade *Stephanosphaerinia* (Chlorophyta) in a basal position in relationship to the clade containing *Balticola, Haematococcus* and *Stephanosphaera* species, and to the clade of *Chloromonas*-like and *Chlorominima* species (Figure 9).

## 4. Discussion

The majority of previous reports on the composition of endolithic communities have focused on extreme regions such as the Negev Desert (Friedmann et al., 1967; Archer et al., 2017), the Atacama Desert (Wierzchos et al., 2006), and the Dry Valleys of Antarctica (Friedmann et al., 1967; Archer et al., 2017).

In contrast, abundant endolithic communities in temperate regions have largely been neglected, with only a few exceptions, e.g., limestone in China (Horath et al., 2006; Tang et al., 2012) or lithic colonization of monumental stones; e.g., Jeronimos Monastery (Lisbon, Portugal) (Ascaso et al., 2002) or Romanesque churches of Segovia (Spain) (de Los Ríos et al., 2009). In this study, we used spectroscopic, microscopic, and molecular analysis to describe endolithic gypsum phototrophs from Sicily, a region typically with a dry temperate climate. By using multiple techniques, we were able to gain a more comprehensive understanding of the distribution, and diversity of these organisms in the region as well as their pigment composition.

It is known that gypsum can form many varieties differing only in their structure (Al-Wakeel, 2002). In this study, three different structures of gypsum were observed. CT-scan images showed that transparent selenites and white crystalline-gypsum from Siculiana Marina (Site 1) and Santa Elisabetta (Site 2) have a similar inner architecture and have a relatively smaller percentage of void space colonized by diverse endolithic communities.

At the remaining two other sites, Monte Perrera (Site 3) and Monte Gibliscemi (Site 4), only light microscopy was used to describe the cyanobacteria, due to the small number of collected samples and low biomass available for metagenomic analysis. At the Monte Perrera site, we identified fewer cyanobacterial species compared to other sites, even though the gypsum found here had the greatest void space, which would in theory provide more space for colonization by microorganisms. The pigmented zones at this site were dominated by a single species. The colonized zones found in the fine-grained gypsum at the Monte Gibliscemi site were only colonized by green algae, and cyanobacteria were virtually absent. These findings suggest that the microbial communities at these sites may be influenced by factors other than the availability of void space in the gypsum; e.g., light availability, pH, or the rate of erosion or weathering.

Differences in the relative abundance of endolithic microorganisms in gypsum of various physical features can be explained by combined effects of environmental factors promoting various ecological interactions within the microbial communities. For example, previous studies have shown that cracks and fissures in calcite allow direct access to sunlight, which then allows deeper colonizations compared to ignimbrite (Crits-Christoph et al., 2016). It was also observed that endolithic communities were less diverse in gypsum with large inner spaces, while more diverse in gypsum with high spatial heterogeneity. This relationship was described as the theory of pore connectivity stating that “low pore connectivity sustain higher microbial diversity because they prevent consumption of resources by more competitive organisms and provide spatial shelter for less competitive organisms” (Ertekin et al., 2020). In other words, the lack of interconnectedness of pores in the gypsum can enhance the diversity of the endolithic community.

In our samples, CT scan results showed that there was almost no difference in the inner microstructures of the two gypsum varieties analysed (Figure 4 and Supplementary Figure 4). However, in terms of light conditions, the transparent selenites should provide better conditions for the growth of photosynthetic microorganisms than do white crystalline gypsum. Indeed, metagenomic analysis has shown that samples of selenites hosted a greater biodiversity (Shannon index; *p* < 0.01), suggesting that the transparency of the gypsum may play a more significant role than microstructure of the substrate in shaping the diversity of these particular endolithic communities.

### 4.1. UV-screening pigments

UV-screening pigments are commonly distributed in endolithic settings, especially in extreme regions that are exposed to increased radiation (Wierzchos et al., 2015; Orellana et al., 2020; Vítek et al., 2020). Among these carotenoids usually dominate as they participated not only in the UV-protection but also in photosynthesis (Maguregui et al., 2012; Jehlička et al., 2016; Osterrothová et al., 2019; Němečková et al., 2020). In our study, carotenoids were detected using Raman spectroscopy in all dominant species (*Nostoc* sp., *G. compacta*, and *G. pleurocapsoides*, orange coccoid algae of the clade *Stephanosphaerinia*). Three Raman bands at ~1,520, 1,155, and 1,008 cm^−1^ are attributed to *ν*_1_ (C=C), *ν*_2_ (C-C) stretching mode, and δ (C-CH_3_) bending mode (Jehlička et al., 2014b). The ν_1_ band wavenumber in some cases can be used for more specific recognition of carotenoids. This band is correlated to the length of the poleyene chain since the wavenumber of *ν*_1_ (C=C) decreases as the number of double bonds in polyene chain increases (Kuzmany, 1980; Withnall et al., 2003; de Oliveira et al., 2010). Precise discrimination of carotenoids is important for taxonomic identification of microorganisms (Edwards et al., 2014; Jehlička et al., 2014a). However, the unambiguous carotenoid discrimination was not possible in the case of cyanobacteria as in biological systems the overall spectrum may also be influenced by other factors, e.g., molecular interactions or stages of life cycles (Collins et al., 2011; Osterrothová et al., 2019). These factors can manifest as shifts in the Raman spectra. In the case of the orange algal stages (*Stephanosphaerinia*) found at Monte Gibliscemi, obtained spectra were rather uniform. Three Raman bands of carotenoids were detected at 1,517, 1,156/55, and 1,005 cm^−1^. From morphologically-similar orange-pigmented coccoid algae isolated from gypsum found in the Atacama Desert, Raman spectra of carotenoids were also obtained at 1,521 and 1,519 cm^−1^ (Wierzchos et al., 2015). These fall in the characteristic area of β-carotene, a common primary carotenoid (Jehlička et al., 2014b).

Besides carotenoids, two other protective pigments were detected—gloeocapsin and scytonemin. Gloeocapsin and scytonemin are dark UV screening pigments, unique for cyanobacteria. Using Raman spectroscopy, these have been previously detected in colonization of various lithic environments, e.g., in limestone (Belgium and Croatia) (Storme et al., 2015), gypsum (Sicily and Poland) (Němečková et al., 2021) or halite (the Atacama Desert, Chile) (Vítek et al., 2014b) In the current study, gloeocapsin was detected particularly in dark-pigmented *Gloeocapsa compacta* found in the upper zones of colonization, with the major Raman bands at 1,671 and 1,571 cm^−1^. Chemically, gloeocapsin is probably a derivative of parietin which has an anthraquinone skeleton (Storme et al., 2015), however, recently, it was also proposed that gloeocapsin could alternatively belong to another class of biomolecules capable of halochromism such as the coumarins, or flavonoids (Lara et al., 2022). Besides the *Gloeocapsa* genus, gloeocapsin was detected in epilithic *Solentia paulocellulare* (Storme et al., 2015) and *Phormidesmis nigrescens* ULC007 (Lara et al., 2022). Currently, the presence of gloeocapsin has only been confirmed in three distinct lineages of cyanobacteria (*Chroococcidiopsidales*, *Chroococcales*, *Leptolyngbyales*) (Lara et al., 2022; Strunecký et al., 2022), and more research is needed to determine the full extent of its occurrence.

On the other hand, scytonemin has been found in many cyanobacteria (Garcia-Pichel and Castenholz, 1991; Dillon et al., 2002; Sorrels et al., 2009; Grant and Louda, 2013; Vítek et al., 2014b; Ekebergh et al., 2015; Gao, 2017). In this study, scytonemin was detected in two species—*Nostoc* sp. and *Gloeocapsopsis pleurocapsoides*, with the most prominent peak at around 1,598 cm^−1^, which corresponds to the ν(CCH) aromatic ring quadrant stretching mode. Scytonemin has been detected by Raman spectroscopy in various lithic ecosystems (usually in extreme regions); e.g., in gypsum (Wierzchos et al., 2015) and halite (Vítek et al., 2012) from the Atacama Desert. In these settings, scytonemin is mainly produced by cyanobacteria in the uppermost zones that are the most irradiated by sunlight (Balskus et al., 2011; Vítek et al., 2014b), which is in accordance with this study. However, Raman spectroscopy has also detected scytonemin in the hypoendolithic colonies underneath the carotenoid-containing zones of gypsum from the Atacama Desert (Wierzchos et al., 2015). This trend may be due to the specific physical features of the colonized gypsum deposits, which allow for some transmission of ultraviolet radiation (Wierzchos et al., 2015).

Besides Raman spectroscopy, the presence of scytonemin was detected indirectly by fluorescence microscopy. Scytonemin absorbs from ~220 to 480 nm, with *in vivo* λ_max_ at 370 nm, which is close to the excitation by DAPI filter (excitation ~365 nm). Due to this, we could observe a lower signal of red autofluorescence of chlorophyll and phycobiliproteins in those zones where scytonemin had accumulated (see Figure 6). A similar trend was also observed in endoliths in halite (Vítek et al., 2014b). In this study, fluorescence analysis shows either a very weak or no red autofluorescence signal of chlorophyll and phycobiliproteins in the black aggregates using GFP (excitation ~450–490 nm). This decrease in red emission was interpreted as the result of scytonemin’s screening effect of the incident green light together with the fact that photosynthetic pigments could be degraded, which would also lead to a decline in the autofluorescence signal (Vítek et al., 2014b). This indirect detection of scytonemin can complement other methods such as Raman spectroscopy. However, this only allows the visualization of the distribution of such compounds in cells, and not the direct detection of scytonemin (or gloeocapsin).

The detection of UV-screening pigments, particularly scytonemin and gloeocapsin, in temperate regions supports previous suggestions about the potential functions of these pigments. Scytonemin synthesis is known to be affected by various environmental stresses, such as temperature, oxidative stress, salinity, and desiccation (Dillon et al., 2002; Vítek et al., 2014b). In addition, Villar et al., have detected scytonemin in gypsum crust, but noted that no scytonemin was detected in rock substrates containing iron oxide, which itself protects against intense solar radiation (Villar et al., 2005). As for gloeocapsin, greater detailed information about its physiological role is still required, but it could be suggested that its biosynthesis may be triggered by various factors, as well.

### 4.2. Diversity of gypsum photrotrophs

#### 4.2.1. Cyanobacteria as the dominant group

Our metagenomic analysis of gypsum from the southern part of Sicily showed the dominance of *Cyanobacteria* over other phyla such as Proteobacteria, Chloroflexi, Bacteroidota, Actinobacteriota, Planctomycetota, and Verrucomicrobiota. Previous studies on metagenomics showed that cyanobacteria significantly dominate as well, e.g., in gypsum from the Atacama Desert (Crits-Christoph et al., 2016). The other detected phyla have also been described from previous studies in calcite and ignimbrite from the hyperarid Atacama Desert (Wierzchos et al., 2015; Crits-Christoph et al., 2016), sandstone, limestone. and granite from the semiarid regions of the Rocky Mountain region (US) (Walker and Pace, 2007), and interestingly, some of them were also detected in an endolithic community associated with the coral *Porites astreoides* (Wegley et al., 2007). Cyanobacteria, Proteobacteria, Actinobacteria, and Acidobacteria were also found in gypsum from the Canadian Arctic (Ziolkowski et al., 2013). The similarities of the diversity on the level of phyla from various lithologies and geographical regions of the planet supports the hypothesis of a global metacommunity. This hypothesis says that endolithic ecosystems are comprised of organisms from a relatively limited reservoir of phylogenetic diversity and that these organisms are specifically adapted to their endolithic habitat (Walker and Pace, 2007). Nevertheless, their relative abundance may vary, for example, Chloroflexi and Proteobacteria were dominant over cyanobacteria in gypsum collected from the Lake St. Martin impact crater (Rhind et al., 2014). In halite from the Atacama Desert, Euryarchaeota were also more abundant compared to cyanobacteria (Wierzchos et al., 2018). As already mentioned, the variances of relative abundance can be related to the physical properties and inner architectures of the inhabited substrates. For instance, a higher taxonomic diversity was observed, in calcite than in ignimbrite (Crits-Christoph et al., 2016). This could be explained by the fact that calcite can form larger fissures and cracks compared to ignimbrite, which would lead to more competition for space and nutrient resources (Crits-Christoph et al., 2016). This also results in different metabolic pathways in these two substrates, genes for ABC transporters and osmoregulation are more diverse in the calcite habitat and genes for secondary metabolites are more abundant in the ignimbrite community (Crits-Christoph et al., 2016). The presence of some genes is also related to the nutrient availability in the environment. For example, no genes for diazotrophy have been detected in calcite and ignimbrite from the Atacama Desert where there are high nitrate accumulations from the atmosphere (Crits-Christoph et al., 2016) compare to. Besides that, the greater abundance of cyanobacteria could be related to the lower water availability, which means decreased photosynthesis and thus less CO_2_ fixation, which is important for the development of heterotrophs (Wierzchos et al., 2018).

In general, in majority of endolithic ecosystems, cyanobacteria are the main primary producers, and the *Chroococcidiopsis* is frequently the major genus (Wierzchos et al., 2018). For example, in the dolomite in south China, nine cyanobacterial major phylotypes were found: *Chroococcidiopsis* sp., *Phormidium autumnale*, *Anabaena oscillarioides*, *Scytonema* sp., *Leptolyngbya* sp., *Calothrix* sp., two *Nostoc* sp., and *Brasilonema octagenarum* (Tang et al., 2012). In gypsum crust from Chott el Jerid, most cyanobacterial phylotypes were related to the genus *Leptolyngbya* within the *Oscillatoriales* and *Nostocales* (Stivaletta et al., 2010). The molecular studies show that the most abundant genomes found in the calcite and ignimbrite were also related to *Chroococcidiopsis* and *Gloeocapsa* (Crits-Christoph et al., 2016). In general, similar compositions have been observed all over the globe in many various substrates (e.g., gypsum crust, Stivaletta et al., 2010), limestone (Ferris and Lowson, 1997; Tang et al., 2012), which could also be explained by the hypothesis of global metacommunity. Also, it has been shown that the overall relative abundance tends to be greater in less extreme environments (Ziolkowski et al., 2013). Nevertheless, the authors acknowledge that it is possible that the higher diversity could be related to the limitations of the molecular methods used (Ziolkowski et al., 2013).

In the current study, we utilized a single cell/filament sequencing technique (Mareš et al., 2015; Kurmayer et al., 2018) to provide a direct link between cyanobacterial morphotypes observed using light microscopy and the results of metagenomic profiling. Such an approach is elaborate, and has only rarely been used in previous studies (Bowers et al., 2022), however, it provides valuable data leading to a better understanding of the microbial communities composition. Another similar, but even more challenging, approach is to link the metagenomic data to barcoded laboratory strains isolated from the same samples (Águila et al., 2021).

The most interesting result was a partial dissection of the cryptic variability in coccoid cyanobacteria that dominated the samples. Surprisingly, the main *Chroococcidiopsis*-like morphotype (Figure 3D) analysed by single-cell sequencing was resolved as *Thermosynechococcaceae* (Figure 8), together with a number of major amplicon sequence variants (ASVs). Some of the remaining frequent ASVs could be linked to the *Chroococcidiopsis sensu stricto* lineage, and even to other lineages of cyanobacteria forming sarcinoid packets of cells (“*Chroococcidopsis*” lineage CC1, *Sinocapsa*, “*Myxosarcina*”). The polyphyly of *Chroococcidiopsis*-like morphotypes have long been known (Cumbers and Rothschild, 2014); however, these taxa are still, for the most part, awaiting formal taxonomic treatment.

The pattern of *Chroococcidiopsis*-like phylotypes found in our study matches those previously reported worldwide from both hot and cold deserts (Bahl et al., 2011), except for the dominant clade in *Thermosynechococcaceae*, for which the morphological information had been missing up to now. Similar organisms were previously sequenced from endolithic limestone communities in the Rocky Mountains and the Mojave Desert, United States (Walker and Pace, 2007; Smith et al., 2014). Another environmental sequence in this clade was erroneously assigned to a true-branching heterocytous cyanobacterium *Loriellopsis cavernicola* (Lamprinou et al., 2011), probably due to laboratory contamination from the sample of calcite rock taken from a cave in Spain.

A complementary line of evidence was provided by single-cell sequencing of the abundant coccoid cyanobacteria containing dark UV-screening sheath pigments, *Gloeocapsa* spp. and *Gloeocapsopsis pleurocapsoides*. All of them clustered in the *Chroococcidiopsidaceae* clade together with a number of frequent amplicon sequence variants and previously published uncultured “*Chroococcidiopsis* sp.” sequences (Figure 8). Reliable sequences of these taxa have never been provided before; thus our results will help to distinguish these eco-physiologically different organisms in future metagenomic studies.

Another interesting finding was the relatively high proportion of *Gloeobacter* in the gypsum samples documented by both light microscopy and amplicon sequencing (Figures 3, 9). While it is known that this basal thylakoid-less cyanobacterium is more common than once thought (Mareš et al., 2013), its global occurrence is still understudied.

The remaining dominant cyanobacteria were classified to *Nostocales* (Nostocaceae) and *Leptolyngbyaceae*. Identity of the dominant *Nostoc* sp. morphotype (Figure 3C) was confirmed by single filament sequencing. However, also in this case, taxa morphologically cryptic to *Nostoc* (*Goleter*; Miscoe et al., 2016 and *Komarekiella*, Johansen et al., 2017) were discovered by the metagenomic analysis along with several other heterocytous cyanobacteria (Figure 8). Surprisingly, the thin filamentous cyanobacteria from *Leptolyngbyaceae* were completely overlooked by microscopy analysis. This is due to the fact that only limited proportion of the samples can be microscopically observed. On the other hand, some of the taxa seen in the microscope (*Microcoleus*, *Petalonema*, *Anathece*) were very scarce or missing in the amplicon sequencing data (e.g., due to better DNA extraction of some species). These findings illustrate the limitations of both approaches.

Considering the community composition, all samples analysed by metagenomic profiling were generally quite similar, and differed only in the proportion of individual groups (Figure 7). As expected, an increased occurrence of cyanobacteria with dark pigmentation (*Nostocales*) was confirmed in the macroscopically dark endolithic layers compared to the green layers dominated by *Thermosynechococcaceae*, in which the sheath pigments are absent.

#### 4.2.2. Endolithic algae

The orange pigmented coccoid algae inhabiting fine-grained gypsum at Site 4 (isolate S14 Sicily) were found to be genetically related to green algae from the *Chlorophyceae* family originated from extreme habitats such as: Antarctic or Arctic freshwater (Jung et al., 2016; Cho et al., 2019), red snow on glacier and green snow fields at King George island (Gálvez et al., 2021), snowfields in Ryder Bay, Antarctic Peninsula (Davey et al., 2019), and Yukidori Valley, Eastern Antarctica (Segawa et al., 2018). Metagenomic sequencing of Arctic gypsum endoliths indicated the presence of few green algal sequences closely matching with *Trebouxia* sp. and *Trichosarcina* sp., known lichenizing symbionts (Ziolkowski et al., 2013). In other study, coccoid chlorophototrophs were responsible for gypsum layered orange to green cryptoendolithic colonization in Atacama, Northern Chile, but its sequences failed to be amplified by the environmental sequencing; likely due to the primer bias (Wierzchos et al., 2015). Another study on chasmoendoliths in Antarctica in granite and gypsum from the Atacama Desert showed the presence of *Trebouxiophyceae* (Yung et al., 2014; Ertekin et al., 2020). Further, known alga found in gypsum (spring associated) was the desmid *Oocardium stratum* (Zygnematophyceae, Streptophyta) (Rott et al., 2010). In our case study, direct DNA extraction isolated from gypsum generated a 18S rDNA sequence corresponding to those from the order of Chlamydomonadales, clade *Stephanosphaerinia*; mostly to genera of *Chloromonas*-like morphology and *Chlorominima* (Figure 9). However, the acquired sequence fragment was too short to allow any closer taxonomic assignment. Some of the related species to our Sicilian algal isolate were shown to form morphologically similar globular-shaped thick-walled cells with cytoplasmic lipid droplets in its life cycle that occupy most of the cell volume. The production of abundant lipid droplets is also correlated with the accumulation of carotenoids (Grünewald et al., 2001), and as mentioned above, carotenoids were also detected in our study by Raman spectroscopy.

## 5. Conclusion

Endolithic ecosystems, which are found in a variety of extreme environments, play a significant role as a model for astrobiology. In this study, we examined gypsum endoliths from Sicily, where gypsum outcrops are quite common. Our results from a metagenomic analysis supported the theory of a global metacommunity, indicating that endolithic communities of a similar general composition can be found across a wide range of environments. In addition to the classical amplicon sequencing approach, single cell sequencing of selected dominant phototrophs allowed us to dissect their phylogenetic relationships and link abundant morphotypes to amplicon sequence variants and Raman spectroscopy data. Unlike in previous studies, the sites we studied were not exposed to extreme solar radiation; yet we still frequently detected UV-protective pigments, scytonemin and gloeocapsin. Overall, our study highlights the diverse and complex nature of endolithic ecosystems, focusing on the previously less studied gypsum substrate and variable colonization of its two forms.

## Data availability statement

The datasets presented in this study can be found in online repositories. The names of the repository/repositories and accession number(s) can be found: NCBI − OQ509475 − OQ509403 + OQ520113.

## Author contributions

JJ, KN, and AC conceived the study. KN, JJ, and AC collected in-field samples. KN carried overall investigation, data interpretation, and writing of the original draft. JM conducted the molecular analysis of cyanobacteria and writing. LP and LN were responsible for the overall molecular analysis of algae and writing. AC and JJ contributed to the Raman analysis and to the writing. FK conducted the CT-scan analysis and writing. JW contributed to the fluorescence analysis and its interpretation. JD, VT, and JŽ assisted with the CT-scan analysis and methodology. AK performed the DNA extraction and validated the PCR protocol. JZ was involved in the 16S rRNA amplification and DNA library preparation. EN prepared the primers and protocols for amplicon analysis and metagenomics. KN, AC, and JJ edited the manuscript. JJ contributed to the conceptualization and provided supervision, resources, and project administration. All authors contributed to the article and approved the submitted version.

## Funding

This project was supported by the Czech Science Foundation (Grant/Award No. 17-04270S and 21-03322S), Ministry of Education, Youth and Sports of the Czech Republic, National Programme of Sustainability I (Grant/Award No. LO1416), Charles University (Grant/Award Nos. UNCE/SCI/006 and UNCE 204069), ALGAMIC (Grant/Award No. CZ.1.05/2.1.00/19.0392). JM was supported by the Czech Science Foundation (GAČR) Project No. 22-06374S to accomplish phylogenetic and taxonomic analysis. JW was thankful for the financial support by the PGC2021-124362NB-I00 grant from MCI/AEI (Spain) and FEDER.

## Conflict of interest

The authors declare that the research was conducted in the absence of any commercial or financial relationships that could be construed as a potential conflict of interest.

## Publisher’s note

All claims expressed in this article are solely those of the authors and do not necessarily represent those of their affiliated organizations, or those of the publisher, the editors and the reviewers. Any product that may be evaluated in this article, or claim that may be made by its manufacturer, is not guaranteed or endorsed by the publisher.

## Footnotes

^1^ http://www.earthmicrobiome.org/protocols-and-standards/16s/
