# Supplementary material for: Gypsum endolithic phototrophs under moderate climate (Southern Sicily): their diversity and pigment composition
Source: Front Microbiol. 2023 Jul 6;14:1175066. doi: 10.3389/fmicb.2023.1175066 (PMC10359912; doi:10.3389/fmicb.2023.1175066)
Supplement: Supplementary file 1 [file Data_Sheet_1.pdf]

Supplementary Material

Gypsum endolithic phototrophs under moderate climate (Southern Sicily): their diversity and pigment composition

Kateřina Němečková*, Jan Mareš, Lenka Procházková, Adam Culka, Filip Košek, Jacek Wierzchos, Linda Nedbalová, Jan Dudák, Veronika Tymlová, Jan Žemlička, Andreja Kust, Jan Zima Jr., Eva Nováková, and Jan Jehlička[1]

*** Correspondence:** Corresponding Author: katerina.nemeckova@natur.cuni.cz

Table S1: Raman bands of the detected pigments.

Table S2: Table of samples used for metagenomics analysis, their lithology, characterization of endolithic colonization and relative abundance of endolithic phototrophs.

Figure S1: Micro-CT 3D reconstructions and selected slices illustrating the inner architecture and void space of gypsum fragments. Samples from Monte Perrera - MP1 (A – B) and MP2 (D – F); and from Monte Gibliscemi MG (G– I). The red and green frames in the 3D images show the position of the displayed slices.

Figure S1: Non-metric Multi-Dimensional Scaling (NMDS) analysis of the diversity among gypsum endolithic samples from two localities in Sicily. Samples from the studied localities are enclosed within convex hulls with centroids marked by asterisks (Siculiana Marina – black, full line, Santa Elisabetta – grey, dashed line). The analysis was based on Bray-Curtis distance matrix inferred from normalized abundances of the amplicon sequence variants (ASVs). The samples from Siculiana Marina exhibited greater overall diversity (they were also greater in number).

Figure S2: Non-metric Multi-Dimensional Scaling (NMDS) analysis of the diversity among gypsum endolithic samples from two different gypsum varieties. Samples from the studied substrates are enclosed within convex hulls with centroids marked by asterisks (selenite – black, full line, crystalline gypsum – grey, dashed line). The analysis was based on Bray-Curtis distance matrix inferred from normalized abundances of the amplicon sequence variants (ASVs). The samples from selenite exhibited greater overall diversity (but they were also significantly greater in number).

Figure S3: Non-metric Multi-Dimensional Scaling (NMDS) analysis of the diversity among gypsum endolithic samples from two differently coloured layers of colonization. Samples from the studied

endolithic layers are enclosed within convex hulls with centroids marked by asterisks (black zone – black, full line, green zone – grey, dashed line). The analysis was based on Bray-Curtis distance matrix inferred from normalized abundances of the amplicon sequence variants (ASVs). The samples from black layers exhibited greater overall diversity.

Site	Sample/ colour of colonisation	Organism	Identified pigment	Detected Raman bands
**Siculiana Marina**	1CF black	*Nostoc* sp.	scytonemin	1712 w, 1633 m, **1600 s**, 1558 m, 1387 w, 1324 w, **1174 ms**, 1098 mw, 1023 w, 755 w, 681 w
		Gloeocapsa sp.	gloeocapsin	1671 m, 1289 m, 480 mw
		Gloeocapsopsis pleurocapsoides	carotenoid	**1516 vs**, 1449 w, 1388 w, 1283 mw, 1189 mw, **1155 ms**, 1004 m, 957 w
**Monte Perrera site**	S12 black	*Gloeocapsa compacta*	gloeocapsin	**1668 s**, **1571 s**, **1424 ms**, 1340 m, **1280 ms**, 1190 m, 465 m, br
		Nostoc sp.	scytonemin	1711 w, 1633 m, **1598 s**, 1555 m, 1381 mw, **1171 ms**, 573 w, 436 w
		Nostoc sp.	carotenoid	**1513 vs**, 1283 w, 1193 m, **1155 s**, 1003 m, 958 w
**Monte Gibliscemi**	S14 orange	*Chlorophyta*	carotenoid	**1517 s**, 1447 w, 1389 w, 1274 w, **1191 ms**, **1156 s**, 1005 m, 964 w

Table S1: Raman bands of the detected pigments.

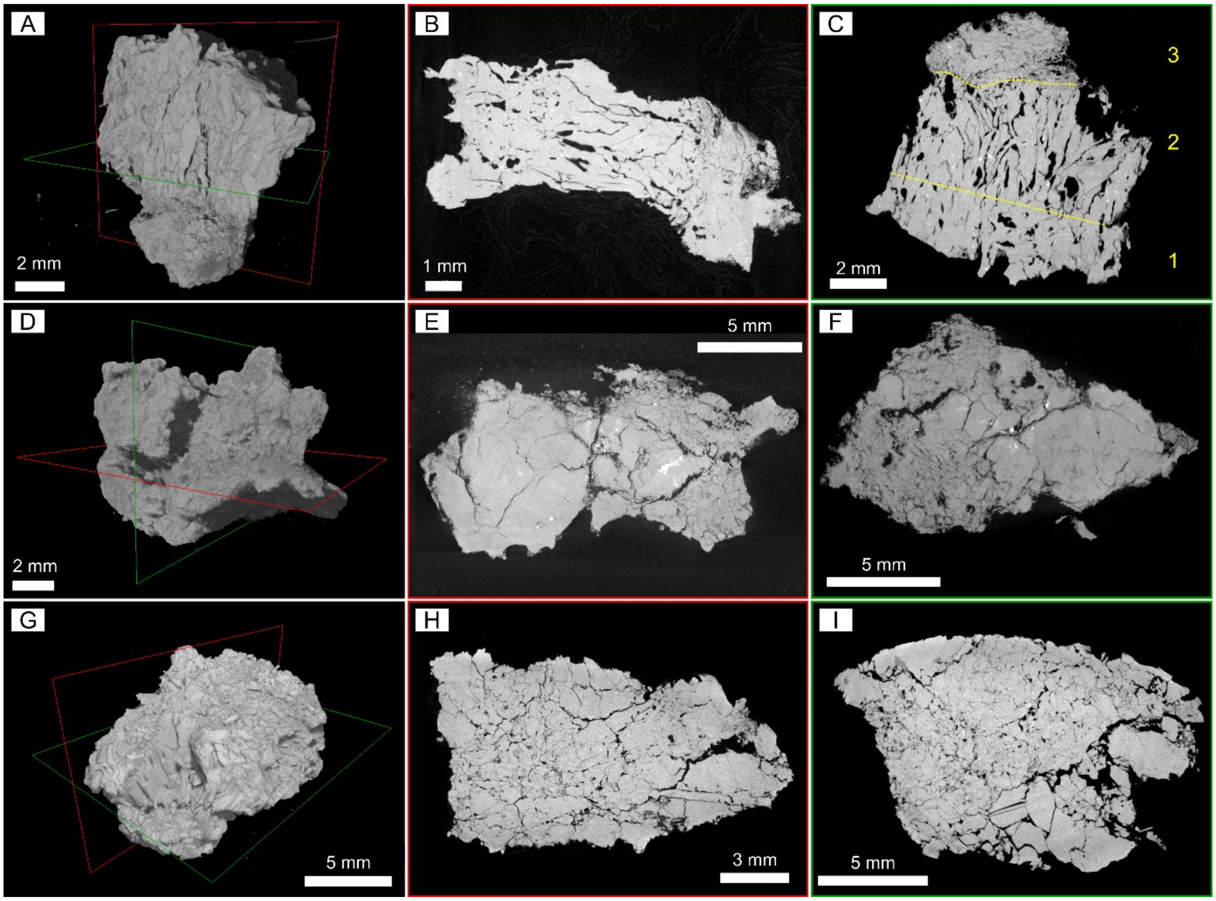

Figure S1: Micro-CT 3D reconstructions and selected slices illustrating the inner architecture and void space of gypsum fragments. Samples from Monte Perrera - MP1 (A – B) and MP2 (D – F); and from Monte Gibliscemi MG (G– I). The red and green frames in the 3D images show the position of the displayed slices.

Site	Lithology	Layer	Sample ID	Relative abundance		
				< 5 %	5-49 %	> 50 %
Siculiana Marina	Selenite	Green	1	*Nostoc* sp., *Microcoleus* sp., *Anathece* sp.	*Chroococcidiopsis* sp., *Chroococus* sp., *Gloeocapsa* sp., *orange-pigmented algae*	-
			2	*Gloeocapsa novacekii, G.rupestris, Nostoc* sp., *Chroococcus* sp.	*Chroococcidiopsis* sp., *Gloeocapsa* sp.	-
			3	*Nostoc* sp. *G. rupestris*	*Gloeocapsa* sp.	*Chroococcidiopsis* sp.,
			4	*Chroococcus, G. Compacta, Nostoc* sp., *Anathece* sp., *Petalonema* sp.	*Gloeocapsa* sp., *G.rupestris*	*Chroococcidiopsis* sp.,
			5		*Chroococcidiopsis* sp., *G. rupestris, G. novacekii*	*Gloeocapsa* sp.
			6	*Chroococcus* sp., *G. compacta*	*Chroococcidiopsis, Gloeocapsa* sp., algae	-
		Black	7		*Chroococcidiopsis* sp., algae	*Nostoc* sp.
			8	*Gloeobacter violaceus, Nostoc* sp., *Chroococcidiopsis* sp., *G. novacekii*	, *Gloeocapsa* sp., *G. rupestris,* algae	*G. compacta*
			9			*Nostoc* sp.
			10	algae		
			11	*Chroococcidiopsis* sp.	*Nostoc* sp., *Gloeocapsa* sp.	*Petalonema* sp.
			12	*Petalonema* sp., algae	*Gloeocapsa* sp.	*Nostoc* sp.
			13	*G. novacekii, Nostoc*	*G. pleurocapsoides, G. rupestris, Gleocapsa* sp. *(bezbarvá)*	*G. compacta*
	White crystalline	Green	14	*Gloeocapsa* sp., *G.compacta, Nostoc* sp.	-	*Chroococcidiopsis* sp.,
		Black	15	*Petalonema* sp., *Chroococcidiopsis* sp., *Nostoc* sp.	*Gloeocapsa* sp., *G.compacta, G. biformis, G. rupestris, Gloeobacter violaceous*	-
		Green	16	*Gloeocapsa* sp., *Nostoc*		*Chroococcidiopsis* sp.,
		Black	17	*Gloeocapsa* sp., *G. novacekii, Nostoc* sp.	*Chroococcidiopsis* sp., *Gloeobacter violaceus, G. rupestris, G. compacta, G. biformis, Petalonema* sp.	-
		Green	18		*Nostoc* sp.	*Chroococcidiopsis* sp.,
		Black	19	*Petalonema* sp., *Chroococcus* sp., *G. novacekii,*	*Chroococcidiopsis* sp., *Nostoc* sp., *G. compacta*	-
		Black	20	*Nostoc* sp., *G. novacekii*	*Gloeocapsa* sp., *G. compacta, G. rupestris, Chroococcidiopsis* sp.	-
Santa Elisabetta	Selenite	green	21	*Petalonema* sp., algae	*Gloeocapsa* sp., algae	*Chroococcidiopsis* sp.,
		black	22		*Gloeocapsa* sp., *Nostoc* sp., *G. biformis,* algae	-
		black	23	*Chroococcidiopsis* sp., algae	*G. rupestris*	*Gloeobacter violaceus*
		black	24	Orange-pigmented algae	*Nostoc* sp.	*Chroococcidiopsis* sp.,
		green	25		*Chroococcidiopsis* sp., *Nostoc* sp.	*Gloeocapsa* sp.
		green	26	*Chroococcidiopsis* sp., algae	-	*Gloeobacter violaceus*
		black	27		*Nostoc* sp., *Chroococcidiopsis* sp., algae	-

Table S2: Table of samples used for metagenomics analysis, their lithology, characterization of endolithic colonization and relative abundance of endolithic phototrophs.

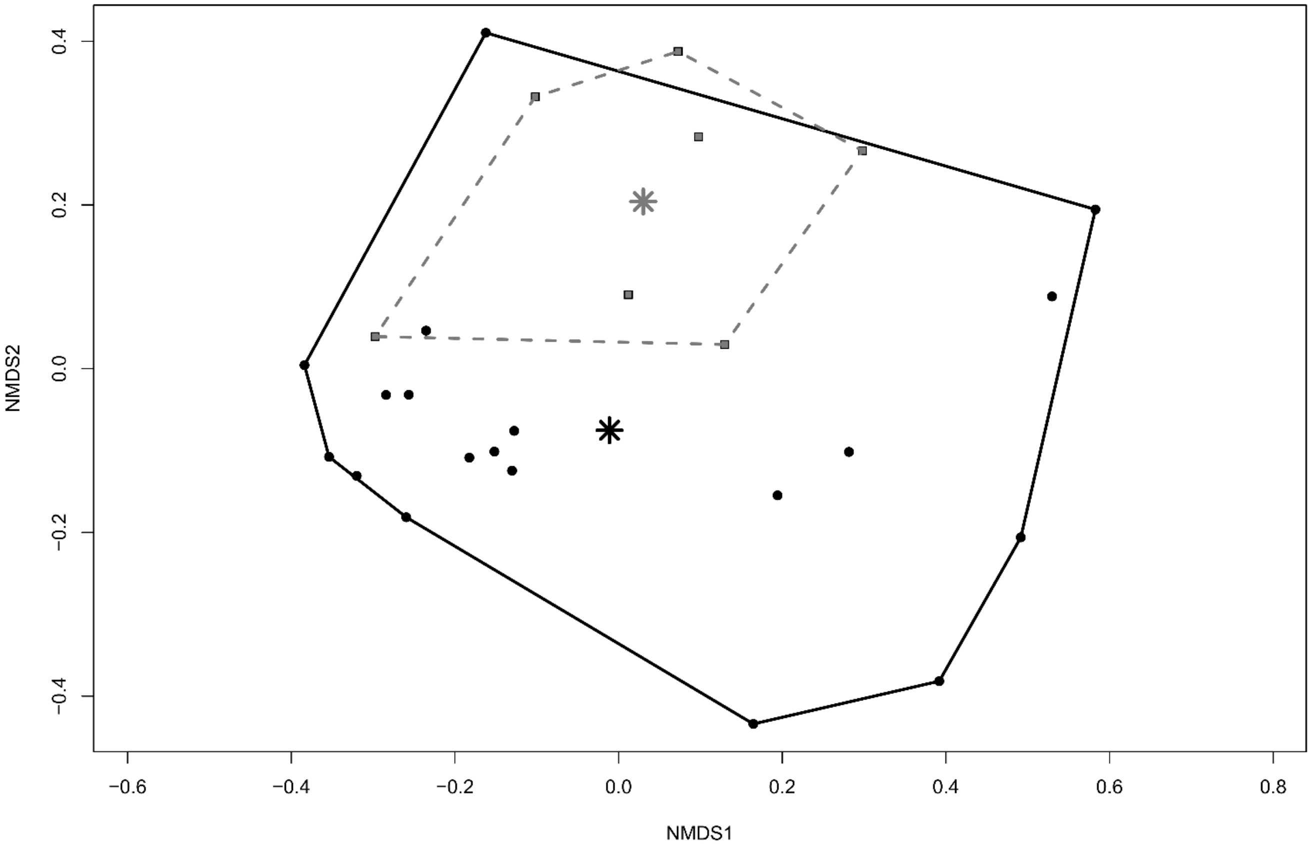

Figure S2: Non-metric Multi-Dimensional Scaling (NMDS) analysis of the diversity among gypsum endolithic samples from two localities in Sicily. Samples from the studied localities are enclosed within convex hulls with centroids marked by asterisks (Siculiana Marina – black, full line, Santa Elisabetta – grey, dashed line). The analysis was based on Bray-Curtis distance matrix inferred from normalized abundances of the amplicon sequence variants (ASVs). The samples from Siculiana Marina exhibited greater overall diversity (they were also greater in number).

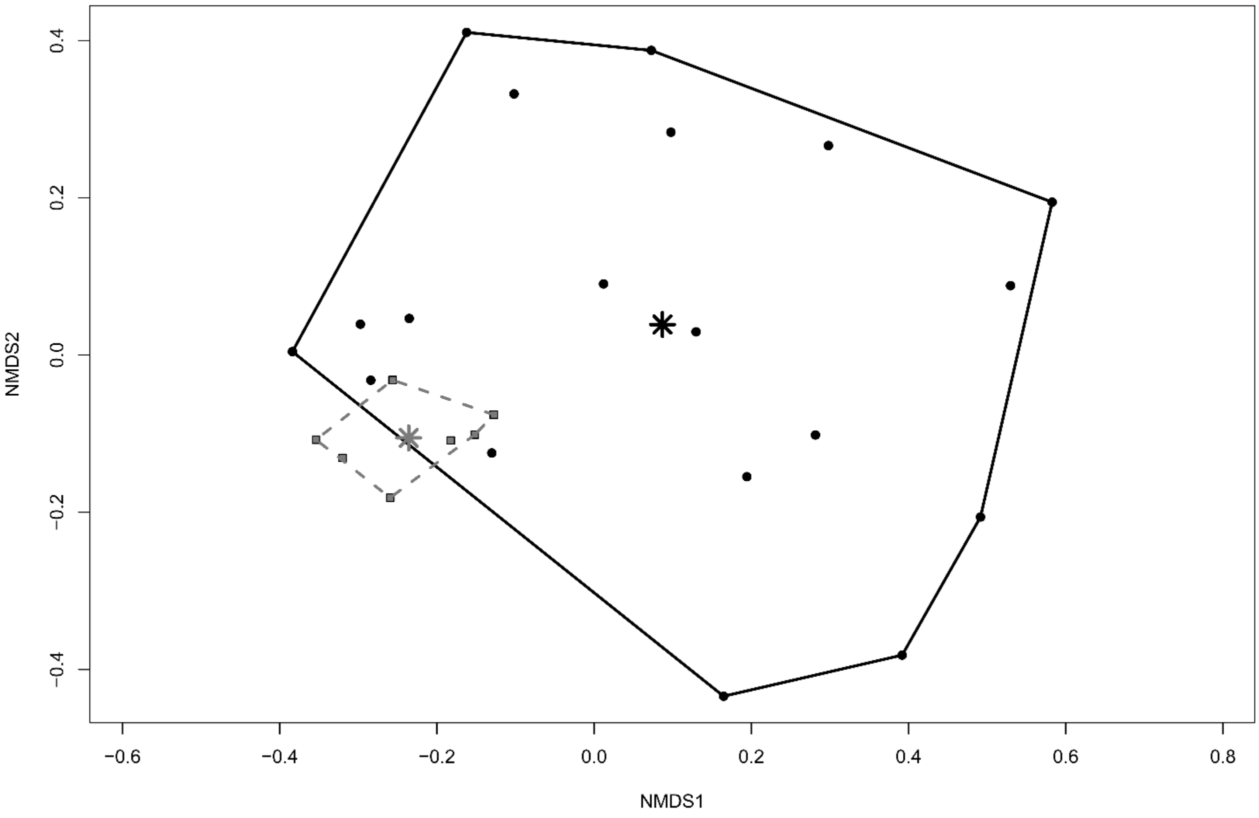

Figure S3: Non-metric Multi-Dimensional Scaling (NMDS) analysis of the diversity among gypsum endolithic samples from two different gypsum varieties. Samples from the studied substrates are enclosed within convex hulls with centroids marked by asterisks (selenite – black, full line, crystalline gypsum – grey, dashed line). The analysis was based on Bray-Curtis distance matrix inferred from normalized abundances of the amplicon sequence variants (ASVs). The samples from selenite exhibited greater overall diversity (but they were also significantly greater in number).

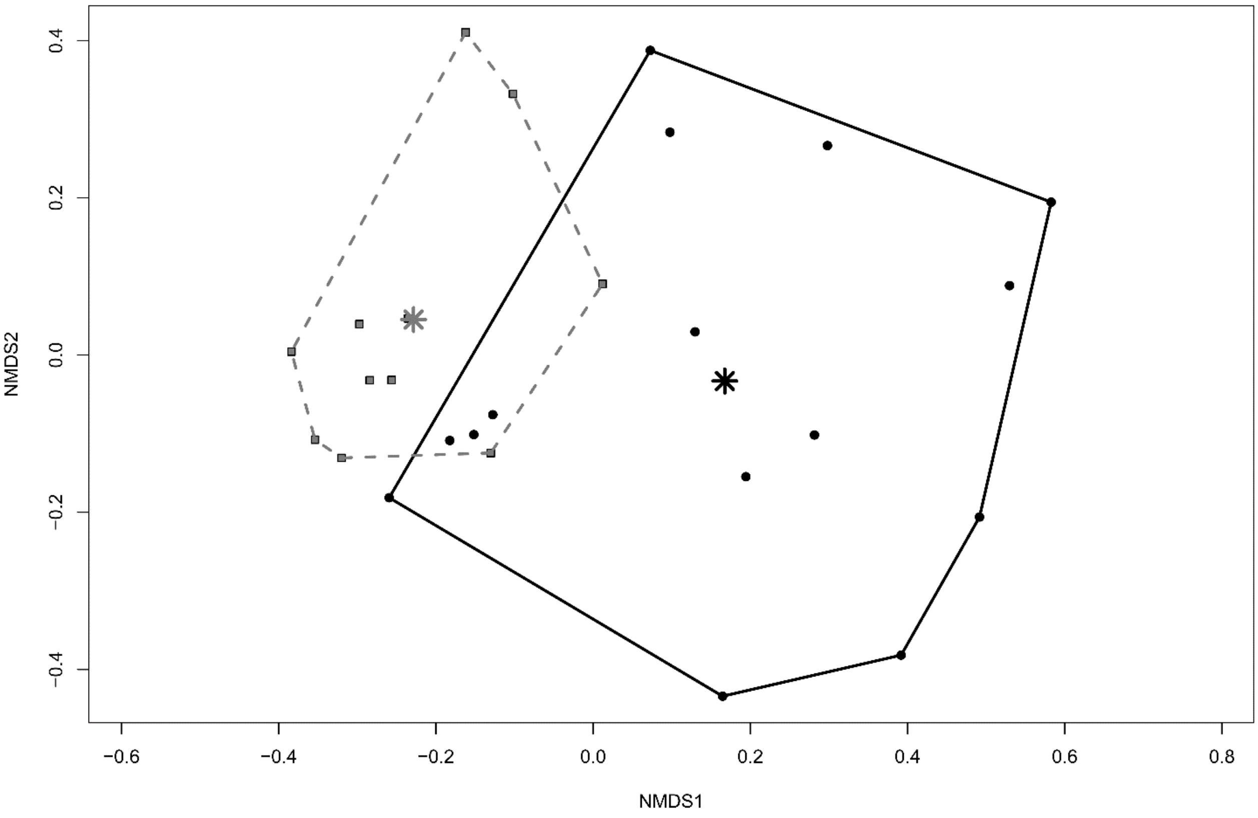

Figure S4: Non-metric Multi-Dimensional Scaling (NMDS) analysis of the diversity among gypsum endolithic samples from two differently coloured layers of colonization. Samples from the studied endolithic layers are enclosed within convex hulls with centroids marked by asterisks (black zone – black, full line, green zone – grey, dashed line). The analysis was based on Bray-Curtis distance matrix inferred from normalized abundances of the amplicon sequence variants (ASVs). The samples from black layers exhibited greater overall diversity.

Accession numbers

Colonizing gypsum under moderate climate: phototrophic endoliths and their pigments

Kateřina Němečková*, Jan Mareš, Lenka Procházková, Adam Culka, Filip Košek, Jacek Wierzchos, Linda Nedbalová, Jan Dudák, Veronika Tymlová, Jan Žemlička, Andreja Kust, Jan Zima Jr., Eva Nováková, and Jan Jehlička[1]

*** Correspondence:** Corresponding Author: katerina.nemeckova@natur.cuni.cz

Cyanobacteria

SUB12910156 Seq1 OQ509403

SUB12910156 Seq2 OQ509404

SUB12910156 Seq3 OQ509405

SUB12910156 Seq4 OQ509406

SUB12910156 Seq5 OQ509407

SUB12910156 Seq6 OQ509408

SUB12910156 Seq7 OQ509409

SUB12910156 Seq8 OQ509410

SUB12910156 Seq9 OQ509411

SUB12910156 Seq10 OQ509412

SUB12910156 Seq11 OQ509413

SUB12910156 Seq12 OQ509414

SUB12910156 Seq13 OQ509415

SUB12910156 Seq14 OQ509416

SUB12910156 Seq15 OQ509417

SUB12910156 Seq16 OQ509418

SUB12910156 Seq17 OQ509419

SUB12910156 Seq18 OQ509420

SUB12910156 Seq19 OQ509421

SUB12910156 Seq20 OQ509422

SUB12910156 Seq21 OQ509423

SUB12910156 Seq22 OQ509424

SUB12910156 Seq23 OQ509425

SUB12910156 Seq24 OQ509426

SUB12910156 Seq25 OQ509427

SUB12910156 Seq26 OQ509428

SUB12910156 Seq27 OQ509429

SUB12910156 Seq28 OQ509430

SUB12910156 Seq29 OQ509431

SUB12910156 Seq30 OQ509432

SUB12910156 Seq31 OQ509433

SUB12910156 Seq32 OQ509434

SUB12910156 Seq33 OQ509435

SUB12910156 Seq34 OQ509436

SUB12910156 Seq35 OQ509437

SUB12910156 Seq36 OQ509438

SUB12910156 Seq37 OQ509439

SUB12910156 Seq38 OQ509440

SUB12910156 Seq39 OQ509441

SUB12910156 Seq40 OQ509442

SUB12910156 Seq41 OQ509443

SUB12910156 Seq42 OQ509444

SUB12910156 Seq43 OQ509445

SUB12910156 Seq44 OQ509446

SUB12910156 Seq45 OQ509447

SUB12910156 Seq46 OQ509448

SUB12910156 Seq47 OQ509449

SUB12910156 Seq48 OQ509450

SUB12910156 Seq49 OQ509451

SUB12910156 Seq50 OQ509452

SUB12910156 Seq51 OQ509453

SUB12910156 Seq52 OQ509454

SUB12910156 Seq53 OQ509455

SUB12910156 Seq54 OQ509456

SUB12910156 Seq55 OQ509457

SUB12910156 Seq56 OQ509458

SUB12910156 Seq57 OQ509459

SUB12910156 Seq58 OQ509460

SUB12910156 Seq59 OQ509461

SUB12910156 Seq60 OQ509462

SUB12910156 Seq61 OQ509463

SUB12910156 Seq62 OQ509464

SUB12910156 Seq63 OQ509465

SUB12910156 Seq64 OQ509466

SUB12910156 Seq65 OQ509467

SUB12910156 Seq66 OQ509468

SUB12910156 Seq67 OQ509469

SUB12910156 Seq68 OQ509470

SUB12910156 Seq69 OQ509471

SUB12910156 Seq70 OQ509472

SUB12910156 Seq71 OQ509473

SUB12910156 Seq72 OQ509474

SUB12910156 Seq73 OQ509475

https://submit.ncbi.nlm.nih.gov/subs/?search=SUB12910156

Algae

OQ520113
